# Serological and faecal markers of irritable bowel syndrome: a systematic review and meta-analysis

**DOI:** 10.1016/j.ebiom.2026.106198

**Published:** 2026-03-06

**Authors:** Grace L. Burns, Freya Roberts, Jasmine A. Wark, Sophie Fowler, Michael P. Jones, Kerith Duncanson, Nicholas J. Talley, Simon Keely

**Affiliations:** a College of Health, Medicine and Wellbeing, The University of Newcastle, Callaghan, Australia; b Immune Health Research Program, Hunter Medical Research Institute, New Lambton Heights, Australia; c NHMRC Centre of Research Excellence in Transforming Gut Health, New Lambton Heights, Australia; d School of Psychological Sciences, Faculty of Medicine, Health and Human Sciences, Macquarie University, Sydney, Australia

**Keywords:** Irritable bowel syndrome, Biomarkers, Blood, Faecal markers, Systematic review

## Abstract

**Background:**

The irritable bowel syndrome (IBS) has long been considered a functional disorder, but recent work has demonstrated clear biological signatures in immune, microbiome and enteric nervous systems of patients with IBS. Despite this new knowledge, there is still no clear biological marker of IBS, with patient symptom reporting and exclusion of organic disease the main criteria for diagnosis. We aimed to perform a systematic review and meta-analysis to identify consistent biomarkers for IBS in serum and stool samples.

**Methods:**

We searched Medline, EMBASE, Cochrane Library, Web of Science and Scopus to obtain all relevant publications published between 1992 and January 2026. Original, peer-reviewed research articles including adults with IBS and healthy or outpatient controls, and/or patients with organic gastrointestinal conditions (e.g. IBD) were included. All articles had quantification of blood and faecal markers between IBS and controls. Descriptive data presented as median and range or median (interquartile range) was converted to mean ± SD. To account for methodological assay differences between studies, standardised mean difference (SMD) with 95% confidence interval was used as the primary outcome measure for the meta-analyses, with a random effects model fitted to the data.

**Findings:**

The search strategy identified 55,444 citations across all databases. 124 studies were included encompassing 14,930 patients with IBS, 7544 healthy/asymptomatic controls and 4317 patients with organic diseases. The top serum discriminators between IBS and healthy controls were TNF-⍺ (13 studies, 1025 controls and 1244 IBS, SMD = 2.74, 95% CI = 0.70, 4.70, *p* = 0.006), IL-6 (13 studies, 736 controls and 1022 IBS, SMD = 1.87, 95% CI = 0.13, 3.61, *p* = 0.035) and IFN-ɣ (4 studies, n = 195 controls, n = 372 IBS, SMD = 2.79, 95% CI = 1.07, 4.51, *p* = 0.002). For faecal markers calprotectin was significantly higher in patients with IBS over controls (11 studies, 1624 controls and 1383 IBS, SMD = 0.75, 95% CI = 0.30, 1.21, *p* = 0.001), while faecal valerate levels were lower in IBS versus controls (4 studies, 290 controls and 488 IBS, SMD = −0.79, 95% CI = −1.48, −0.11, *p* = 0.02). For discriminating IBS overall from organic diseases, serum albumin (4 studies, 282 IBS and 312 organic, SMD = 2.15, 95% CI = 0.20, 4.11, *p* = 0.031) and faecal calprotectin (16 studies, 1591 IBS and 1685 organic, SMD = −1.13, 95% CI = −1.51, −0.75, *p* < 0.0001) were significantly different. In discriminating IBS subtypes from controls, only diarrhoeal IBS (IBS-D) could be distinguished by albumin (3 studies, 248 controls and 219 IBS-D, SMD = −0.39, 95% CI = −0.68, −0.11, *p* = 0.007) and IL-6 (4 studies, 153 IBS-D and 169 controls, SMD = 2.53, 95% CI = 0.86, 4.21, *p* = 0.003). Heterogeneity across the studies ranged from moderate to high, but few overly influential studies were identified between comparisons.

**Interpretation:**

Patients with IBS exhibit increased peripheral cytokine levels that are consistent with reports of increased epithelial permeability and may be important in distinguishing subgroups of IBS patients. Patients with IBS also demonstrated higher faecal calprotectin levels than healthy individuals, although these levels were still significantly lower than patients with organic diseases. Similarly, patients with IBS-D have lower serum albumin levels compared to healthy controls, while patients with organic disease had lower levels compared to patients with IBS, irrespective of subtype. There are clear biological signatures at play in IBS patients that may be useful clinically in establishing IBS diagnosis and may indicate the mechanisms of disease symptoms.

**Funding:**

10.13039/501100000925National Health and Medical Research Council Centre for Research Excellence in Digestive Health (NJT, SK) G180219.

**Research in context**

**Evidence before this study**

While long considered a functional disorder, more recent work in the field has suggested abnormalities in factors of the immune, microbiome and enteric nervous systems of patients with IBS. However, the findings have often been conflicting, largely due to heterogeneity in the presentation and course of IBS itself, as well as differences in diagnostic criteria, study cohort sizes and methodologies. As such, no clear biological indicator(s) of IBS have been confirmed and incorporated into the diagnostic work-up of IBS.

**Added value of this study**

This study encompasses 124 studies and a total of 26,791 participants, with meta-analyses conducted for suitable factors. This work provides quantitative evidence that there are distinct factors in the peripheral blood and faeces of patients with IBS in comparison to the healthy population. Of particular interest, this study demonstrates a strong signature of elevated pro-inflammatory cytokines, including TNF-⍺, IL-6 and IFN-ɣ in IBS patients compared to controls, and the potential for faecal calprotectin levels to differentiate health from patients with IBS and from patients with organic disease. Further, this study identified patterns specific to the IBS diarrhoea subtype, including lower albumin levels and higher circulating IL-6 that may offer new insights into underlying mechanisms.

**Implications of all the available evidence**

The combined evidence from this study and previous literature supports IBS as a condition with measurable biological alterations in immune and inflammatory pathways. With further characterisation, these findings continue to challenge the traditional view as a purely functional disorder and support the potential for biomarker-based diagnostic and treatment stratification tools. Clinically, such an approach would reduce reliance on symptom-based criteria and exclusionary testing, allowing for earlier and accurate diagnosis and tailored management.

## Introduction

The irritable bowel syndrome (IBS) is classified as a disorder of gut-brain interaction (DGBI), a group of heterogeneous gastrointestinal conditions which have previously been described as ‘functional’ disorders. Like most other DGBIs, IBS is diagnosed based on meeting a set of symptom criteria (the Rome criteria)^1^ relating to abdominal pain and altered bowel habits, with the exclusion of organic diseases such as inflammatory bowel disease (IBD). Patients with IBS are further subtyped based on the predominant bowel-habit symptom into diarrhoea (IBS-D), constipation (IBS–C), mixed (IBS-M) or indeterminate.^2^ While the cause of IBS is not known, up to 20% of cases develop after an episode of acute gastroenteritis (post-infectious, PI-IBS).^2^ As IBS diagnosis is largely based on symptom profiles, IBS has conventionally been considered to have no overt structural pathology or biochemical signature. However, recent work in the field has identified a range of sub-clinical inflammatory abnormalities in patients, suggestive of altered immune homoeostasis.^3^ This has led to research efforts to identify biological signatures or “biomarkers” for IBS, which may provide a more reliable and rapid diagnostic approach for IBS.

Biomarkers are attractive targets for diagnosis, staging, monitoring and measuring disease risk in some organic gastrointestinal (GI) conditions, including colorectal cancer^4^ and inflammatory bowel disease (IBD).^5^ A biomarker can be an abnormal physiologic mechanism, a gene, protein or metabolite; and its utility is reliant on its objective measure of a biological process or response to pathogen.^6^ With these prerequisites in mind, IBS represents a condition for which identifying objective and specific biomarkers is very challenging due to the heterogeneity of disease presentation. Current reliance on patient symptom self-report to physicians for diagnosis using the Rome criteria^1^ highlights the need for objective measures of disease. Consensus in the field currently suggests that a panel of markers, as opposed to a single marker, will be necessary to discriminate IBS.^7^, ^8^, ^9^ The discovery of validated biomarkers with a capacity to differentiate disorders of gut-brain interaction (DGBIs) from organic GI disease is important in reducing the number of costly diagnostic tests currently required due to the symptom overlap between conditions. In addition, the capacity to objectively identify IBS subtypes is crucial in predicting therapeutic response with the aim of improving patient quality of life (QoL). As previously reported by Camilleri,^10^ given the phenotypic diversity of IBS and other DGBIs, the most promising biomarkers are those which identify biological or psychological features of DGBI subsets that can be manipulated by available therapeutics. However, to date proposed biomarker panels have not successfully translated into routine patient management. Although a number of peripheral and faecal factors have been proposed to differentiate IBS from health and/or other GI disease, many of these proposed markers have given inconsistent results and the utility and reproducibility of these factors has not comprehensively been systematically reviewed. As such, this systematic review aimed to answer the questions ‘what blood and faecal factors are currently proposed to quantitatively differentiate IBS from health and/or other GI diseases?’ and ‘do these markers effectively discriminate between IBS and health, as well as between IBS and organic gastrointestinal diseases?’.

## Methods

### Search strategy

A systematic literature search was performed in Medline, EMBASE, Cochrane Library, Web of Science and Scopus to obtain all relevant publications published between 1992 and January 2026. This review adhered to the Preferred Reporting Items for Systematic Reviews and Meta-Analyses (PRISMA) guidelines (Supplementary Table S1) and was registered in the PROSPERO systematic review database (CRD42024502876, May 2024). The search strategy used is outlined in Supplementary Table S2 and encompassed combinations of terms describing IBS, blood and faecal factors and biomarkers. The terms were searched using limits that included only articles published in the English language. Handsearching of reference lists of recent reviews on IBS was conducted to ensure all relevant articles were included.

### Study selection

Four reviewers (GLB, FR, SK, JAW) independently screened title and abstracts for relevance to the topic in Covidence (covidence.org) using manual screening. The remaining articles underwent full text screening for suitability. Original, peer-reviewed research articles including adults (aged older than 18 years) with a diagnosis of IBS and healthy or outpatient controls, and/or patients with organic gastrointestinal conditions (e.g. IBD) were suitable for inclusion. Included articles must have included quantification of blood and faecal markers between IBS and controls. Paediatric studies, reviews, theses and case studies were excluded. Full exclusion and inclusion criteria are outlined in Supplementary Table S3. Conflicts at both the title/abstract and full text screening stages were resolved by the fourth reviewer (JAW).

### Data extraction

Data manually extracted included publication, country, the IBS diagnostic criteria used, sample size, participant sex and age, disease subtype (where reported), control population type(s), sample type and collection details (where reported), as well as the overall study aim, analysis methods (including manufacturer/kit details where reported) and values for each reported peripheral/faecal factor between the IBS and control groups(s), as well as subtypes where available. For papers where the summary statistics were not reported in the main text, but were included visually in a clear form that could be extracted from the figures, the metaDigitise package (v1.0.1)^11^ in Rstudio (v2024.09.0) using R (v4.4.1) was used to approximate mean and SD. Where this approach was used, calculated means were compared manually to the graphs to ensure the extracted values were as accurate as possible. This study focused on unstimulated levels of assayed factors compared between groups, so in studies where there was an intervention or *ex vivo* assessment of factors in response to stimuli or similar, only baseline (unstimulated) data were extracted if available.

### Meta-analysis and statistics

If required prior to analysis, descriptive data presented as median and range or median (interquartile range) were converted to mean ± SD using Meta-Converter, now known as Meta-Analysis Accelerator^12^ (https://ma-accelerator.com/conversions). Studies that only reported descriptive statistics by IBS subtype were combined into a single overall IBS group sequentially using Cochrane's formula^13^ (https://www.statstodo.com/CombineMeansSDs.php) for IBS compared to healthy/asymptomatic and organic controls, with an assumption of non-normal distribution for all comparisons.

For factors that were assessed in 3 or more unrelated studies with quantitative data reported, a meta-analysis was performed using the metafor package (v4.6.0)^14^ in Rstudio (Supplementary Methods). To account for differences in units reported, kits/reagents/instruments used for assessment and general laboratory variation between studies, standardised mean difference (SMD) with 95% confidence interval was used as the primary outcome measure for the meta-analyses,^15^ with a random effects model (restricted maximum likelihood estimator, REML)^16^ fitted to the data. For SMD, values < 0.5 were considered small, 0.5–0.8 medium and >0.8 large. The REML model was chosen as most appropriate for this study due to observed heterogeneity of effect sizes, study approaches and disease variation. Heterogeneity was quantified using the I^2^ statistic,^17^ where >50% was considered moderate and >75% considered substantial variability between studies. For factors with substantial heterogeneity, meta-regression was conducted in metafor to assess the potential contribution of moderating factors including the diagnostic criteria used, total study participant size, percentage of female IBS patients and geographical region of the study. For these analyses, R^2^ is the percentage of heterogeneity the factor in question accounts for. Where moderating data were not reported for a factor, these factors were not analysed due to the relatively small number of studies included in the meta-analysis. Studentized residuals and Cook's distance were used to assess whether studies were potential outliers/influential in the model.^18^ For significant findings where an outlier was identified by the studentized residuals and/or Cook's distances tests, and there would be more than 3 studies remaining for analysis, a leave-one-out analysis was performed to assess the robustness of the results. For analyses with 10 or more studies included, the Egger's regression test^19^ using standard error of the observed outcomes as the predictor was used to assess funnel plot asymmetry. Where the Egger's test suggested significant publication bias, the Duval and Tweedie trim-and-fill method and the R0 estimator was used to assess bias-adjusted effect sizes. The alpha level of significance was set at *p* < 0.05. Summary statistics and figures of study characteristics were analysed in GraphPad Prism (GraphPad software LLC, v10.0.3). Given the assessment of both standard and high-sensitivity (hs-CRP) C- reactive protein (CRP) have been shown to be comparable^20^ and SMD was used as the primary outcome measure, studies using either method were meta-analysed together.

### Ethical approval

Given this is a systematic review and meta-analysis, this study was conducted using publicly available data published in previous studies and thus did not require ethical approval.

### Role of the funders

This work was supported by the National Health and Medical Research Council Centre for Research Excellence in Digestive Health. The funding source played no role in the study design or analysis.

## Results

### Characteristics of the included studies

**Fig. 1:**
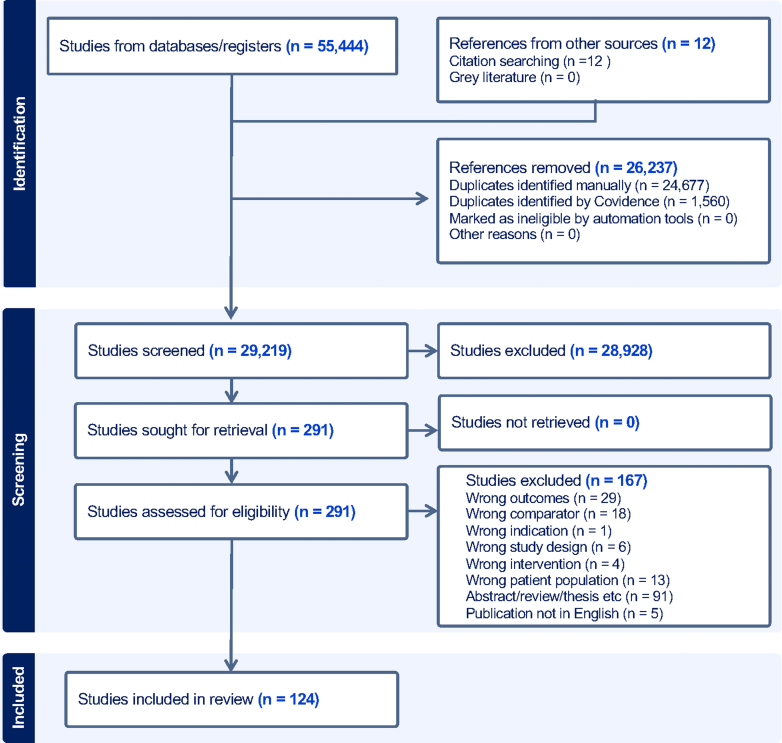
Preferred reporting items for systematic review (PRISMA) literature search flowchart. Flow diagram outlining the process for identification of studies deemed suitable for inclusion in the overall systematic review.

The search was last conducted in January 2026 and identified 55,444 references across all databases, of which 26,237 were duplicates and excluded. Of the remaining 29,219 references identified by the search strategy, and an additional 12 references identified by hand searching, 28,928 were excluded based on title and abstract, with 291 selected for full text review. 167 studies were excluded, and 124 met the criteria for inclusion in the review^21^, ^22^, ^23^, ^24^, ^25^, ^26^, ^27^, ^28^, ^29^, ^30^, ^31^, ^32^, ^33^, ^34^, ^35^, ^36^, ^37^, ^38^, ^39^, ^40^, ^41^, ^42^, ^43^, ^44^, ^45^, ^46^, ^47^, ^48^, ^49^, ^50^,^51^, ^52^, ^53^, ^54^, ^55^, ^56^, ^57^, ^58^, ^59^, ^60^, ^61^, ^62^, ^63^, ^64^, ^65^, ^66^, ^67^, ^68^, ^69^, ^70^,^71^, ^72^, ^73^, ^74^, ^75^, ^76^, ^77^, ^78^, ^79^, ^80^, ^81^, ^82^, ^83^, ^84^, ^85^, ^86^, ^87^, ^88^, ^89^, ^90^, ^91^, ^92^, ^93^, ^94^, ^95^, ^96^, ^97^, ^98^, ^99^, ^100^,^101^, ^102^, ^103^, ^104^, ^105^, ^106^, ^107^, ^108^, ^109^, ^110^, ^111^, ^112^, ^113^, ^114^, ^115^,^116^, ^117^, ^118^, ^119^, ^120^, ^121^, ^122^, ^123^, ^124^, ^125^, ^126^, ^127^, ^128^, ^129^, ^130^, ^131^, ^132^, ^133^, ^134^, ^135^, ^136^, ^137^, ^138^, ^139^, ^140^, ^141^, ^142^, ^143^, ^144^ (Fig. 1). 78 studies had extractable data on peripheral factors alone, 26 on faecal factors alone and 20 assessed both peripheral and faecal factors with data suitable for extraction (Supplementary Table S4).

The 124 included studies encompassed 14,930 patients with IBS, 7544 healthy/asymptomatic controls and 4317 patients with organic disease. In the IBS cohort, 8721 were reported as having the diarrhoeal subtype (IBS-D), 1824 were constipated (IBS-C), while 1935 patients with IBS were reported as having the mixed subtype (IBS-M) and 325 had unknown IBS (IBS-U). No subtype was reported for 2112 patients. In addition, 77 patients with IBS were reported to have post-infectious onset IBS (PI-IBS). Regarding organic gastrointestinal diagnoses, the included studies encompassed 1582 Crohn's disease (CD), 1571 ulcerative colitis (UC) and 547 other or unspecified IBD cases. There were also 279 patients with coeliac disease, 27 with GI infections and 198 with other or unspecified GI conditions. 9487 patients with IBS (68.63% of participants in studies reporting sex), 3919 controls (58.95%) and 1879 participants with organic GI disease (52.77%) in the included studies were female. Sex was not reported for 1106 IBS, 896 control or 756 participants with organic GI disease.

The median publication year was 2019 (range 1995–2025) (Supplementary Figure S1A), and studies were conducted across 33 countries, with most conducted in China, Sweden, United States of America, Turkey, the Netherlands, United Kingdom, Iran, Italy, India, Egypt, Norway and Australia (Supplementary Figure S1B). Rome III was the most prevalent diagnostic criteria for IBS used (n = 55 studies), followed by Rome IV (n = 33) and Rome II (n = 23) (Supplementary Figure S1C).

### There is a peripheral inflammatory cytokine signature in IBS over healthy/asymptomatic controls

**Fig. 2:**
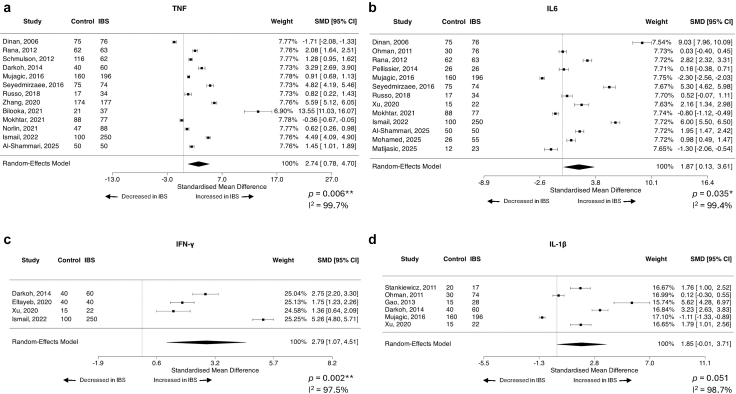
Forest plots of peripheral cytokines that differentiate IBS patients from healthy/asymptomatic controls. Standardised mean difference (SMD) with 95% confidence intervals were assessed using a random effects model for studies reporting quantitative systemic levels of (a) TNF-⍺ (n = 13 studies, n = 1025 controls, n = 1244 IBS), (b) IL-6 (n = 13 studies, n = 736 controls, n = 1022 IBS), (c) IFN-ɣ (n = 4 studies, n = 195 controls, n = 372 IBS) and (d) IL-1β (n = 6 studies, n = 280 controls, n = 397 IBS) between IBS subjects and healthy or asymptomatic control patients. Heterogeneity of the included publications was tested using the I^2^ statistic. Forest plots presented as IBS over control, where a positive SMD means the assessed factor is increased in IBS over control.

For comparison between IBS and healthy or asymptomatic control populations, the following peripheral factors were assessable by meta-analysis: serotonin (5-HT), albumin, zonulin, tumour necrosis factor alpha (TNF-⍺), interleukin (IL)-6, anti-cytolethal distending toxin B (CtdB), anti-vinculin antibodies, CRP, interferon gamma (IFN-ɣ), IL-10, IL-1β, IL-8, leptin, cortisol, and norepinephrine. The findings for all are reported in (Table 1). Thirteen studies^25^^,^^34^^,^^55^^,^^57^^,^^62^^,^^66^^,^^75^^,^^76^^,^^81^^,^^99^^,^^120^^,^^123^^,^^135^ (encompassing 1025 controls and 1244 IBS) assessed on the peripheral levels of TNF-⍺, finding levels were increased in IBS compared to controls (SMD = 2.874, 95% CI = 0.70, 4.70, *p* = 0.006) (Fig. 2a). The heterogeneity of the included studies was considerable (I^2^ = 99.7%), and both the studentized residuals and Cook's distances tests identified one study^135^ that could be considered overly influential. Neither diagnostic criteria, total participant number, percentage of females in cohort nor geographical region (R^2^ = 0% for all) explained the substantial heterogeneity observed across studies. The removal of the outlying study from the analysis still identified increased TNF-⍺ in IBS over control (SMD = 1.94, 95% CI = 0.69, 3.18, *p* = 0.002). Egger's test suggested the possibility of publication bias (*p* < 0.0001), however, the trim-and-fill method estimated that there were no studies missing from the left side of the funnel plot, suggesting the asymmetry is not due to publication bias alone (Supplementary Figure S2A).

**Table 1:** IBS versus control, all possible datasets meta-analysed.

Factor	Blood or faecal	Studies (n)	Control (n)	IBS (n)	Average SMD (95% CI)	*p* value	I^2^ statistic	Study references
Serotonin	Blood	7	409	465	1.72 (−1.88, 5.32)	0.35	99.81%	Guo, 2018^109^; Yu, 2016^37^; Barandouzi, 2022^139^; Chojnacki, 2022^128^; Mujagic, 2022^77^; James, 2023^98^; Chojnacki, 2024^127^
Albumin	Blood	5	308	326	−0.21 (−0.48, 0.05)	0.11	57.84%	Boga, 2016^134^; Makhlouf, 2021^85^; Yao, 2023^39^; Borecki, 2021^133^; Huang, 2024^101^
Zonulin	Blood	5	350	384	0.54 (−0.10, 1.18)	0.101	93.53%	Singh, 2019^54^; Barbaro, 2020^138^; Talley, 2020^50^; de Faria, 2021^121^; Rezazadegan, 2022^63^)
TNF-⍺	Blood	13	1025	1244	2.74 (0.7, 4.70)	0.006∗∗	99.70%	Dinan, 2006^120^; Rana, 2012^66^; Schmulson, 2012^57^; Darkoh, 2014^123^; Mujagic, 2016^76^; Seyedmirzaee, 2016^55^; Russo, 2018^62^; Zhang, 2020^34^; Bilooka, 2021^135^; Mokhtar, 2021^81^; Norlin, 2021^75^; Ismail, 2022^99^; Al-Shammari, 2025^25^
IL-6	Blood	13	736	1022	1.87 (0.13, 3.61)	0.035∗	99.4%	Dinan, 2006^120^; Ohman, 2012^73^; Rana, 2012^66^; Pellissier, 2014^70^; Mujagic, 2016^76^; Seyedmirzaee, 2016^55^; Russo, 2018^62^; Xu, 2020^40^; Mokhtar, 2021^81^; Ismail, 2022^99^; Al-Shammari, 2025^25^; Mohamed, 2025^22^; Matijasic, 2025^23^
Anti-cytolethal distending toxin B	Blood	4	376	2770	0.34 (−0.20, 0.88)	0.221	91.64%	Pimentel, 2015^69^; Talley, 2019^51^; Hanevik, 2022^105^; Barros, 2025^24^
Anti-vinculin antibodies	Blood	3	555	2789	0.22 (−23, 0.67)	0.340	93.67%	Pimentel, 2015^69^; Talley, 2019^51^; Barros, 2024^24^
C-reactive protein	Blood	13	686	953	0.50 (−0.43, 1.43)	0.293	98.34%	Boga, 2016^134^; Manolakis, 2017^84^; Weaver, 2018; Bilooka, 2021^135^; Borecki, 2021^133^; Makhlouf, 2021^85^; Guven, 2022^107^; Chojnacki, 2022^128^; Sarhan, 2023^59^; Chojnacki, 2024^127^; Roth, 2024^21^; Al-Shammari, 2025^25^; Tuncel, 2025^28^
IFN-ɣ	Blood	4	195	372	2.79 (1.07, 4.51)	0.002∗∗	97.48%	Darkoh, 2014^123^; Eltayeb, 2020^116^; Xu, 2020^40^; Ismail, 2022^99^
IL-10	Blood	15	899	1195	0.48 (−2.24, 3.21)	0.728	99.85%	Dinan, 2006^120^; Ohman, 2012^73^; Rana, 2012^66^; Stankiewicz, 2011^53^; Schmulson, 2012^57^; Gao, 2013^111^; Darkoh, 2014^123^; Mujagic, 2016^76^; Zhu, 2019^32^; Eltayeb, 2020^116^; Xu, 2020^40^; Bilooka, 2021^135^; Ismail, 2022^99^; Lui, 2022^87^; Matijasic, 2025^23^
IL-1β	Blood	6	280	397	1.85 (−0.01, 3.71)	0.051	98.68%	Stankiewicz, 2011^53^; Ohman, 2012^73^; Gao, 2013^111^; Darkoh, 2014^123^; Mujagic, 2016^76^; Xu, 2020^40^
IL-8	Blood	6	430	479	4.22 (−0.29, 8.72)	0.067	99.82%	Dinan, 2006^120^; Mujagic, 2016^76^; Seyedmirzaee, 2016^55^; Russo, 2018^62^; Xu, 2020^40^; Mokhtar, 2021^81^
Leptin	Blood	3	117	156	−0.16 (−1.06, 0.73)	0.72	90.51%	Semnani, 2009^56^; Liu, 2018^88^; Russo, 2018^62^
Cortisol	Blood	4	122	121	1.20 (−1.79, 4.18)	0.433	98.41%	Gorard, 1995^110^; Dinan, 2006^120^; Pellissier, 2014^70^; Weaver, 2018^41^
Norepinephrine	Blood	3	118	126	0.52 (−0.82, 1.85)	0.45	95.32%	Pellissier, 2014^70^; Barandouzi, 2022^139^; James, 2023^98^
Butyrate	Faeces	6	334	600	−0.29 (−0.60, 0.01)	0.06	71.76%	Mujagic, 2016^76^; Tian, 2019^45^; Zhang, 2019^35^; Xu, 2020^40^; Wang, 2022^42^; Gargari, 2023^30^
Calprotectin	Faeces	11	1624	1383	0.75 (0.30, 1.21)	0.001∗∗	94.98%	Foell, 2007; Mujagic, 2016^76^; Fu, 2017; Melchior, 2017; Thorsvik, 2017; Gacesa, 2021; de Graaf, 2022; Sarhan, 2023^59^; Chojnacki, 2022^128^; Chojnacki, 2024^127^; Huong, 2024
Valerate	Faeces	4	290	488	−0.79 (−1.48, −0.11)	0.02∗	93.06%	Mujagic, 2016^76^; Zhang, 2019^35^; Xu, 2020^40^; Gargari, 2023^30^
Propionate	Faeces	4	219	330	0.08 (−0.09, 0.26)	0.346	0.00%	Mujagic, 2016^76^; Tian, 2019^45^; Zhang, 2019^35^; Xu, 2020^40^; Wang, 2022^42^
Chromogranin A	Faeces	3	1237	453	1.10 (−0.73, 2.93)	0.238	99.34%	Mujagic, 2016^76^; Gacesa, 2021^112^; Sundin, 2018^52^)
Acetate	Faeces	4	219	329	−0.24 (−0.65, 0.18)	0.267	70.55%	Mujagic, 2016^76^; Tian, 2019^45^; Xu, 2020^40^; Wang, 2022^42^

Positive SMD means factor is increased in IBS over control, negative SMD means factor is decreased in IBS over control. ∗*p* < 0.05; ∗∗*p* < 0.01.

IL-6 was also significantly increased in IBS compared to healthy/asymptomatic control subjects across thirteen studies^22^^,^^23^^,^^25^^,^^40^^,^^55^^,^^62^^,^^66^^,^^70^^,^^73^^,^^76^^,^^81^^,^^99^^,^^120^ with 736 controls and 1022 IBS patients (SMD = 1.87, 95% CI = 0.13, 3.61, *p* = 0.035) with considerable heterogeneity (I^2^ = 99.4%) (Fig. 2b). Neither total participant number, IBS diagnostic criteria, percentage of females in cohort nor geographical region explained the heterogeneity across studies (R^2^ = 0% for all). No studies were identified as likely overly influential, however Egger's test suggested potential funnel plot asymmetry (*p* = 0.006) (Supplementary Figure S2B). The trim-and-fill method estimated that there were no studies missing from the left side of the funnel plot, suggesting the asymmetry is not due to publication bias alone.

Only four studies^40^^,^^99^^,^^116^^,^^123^ assessing IFN-ɣ were suitable for meta-analysis, however this cytokine was also significantly increased in IBS compared to controls (n = 195 controls, n = 372 IBS, SMD = 2.79, 95% CI = 1.07, 4.51, *p* = 0.002) with considerable heterogeneity (I^2^ = 97.5%) (Fig. 2c). Total number of participants accounted for 96.73% of the heterogeneity across studies (I^2^ = 52.64%, QM = 52.79, *p* < 0.0001), with a small but significant increase in effect size associated with additional participants (estimated coefficient β = 0.01, 95% CI 0.01, 0.02, *p* < 0.0001). Geographical region did not account for significant heterogeneity (R^2^ = 0%). Cook's residuals did not identify any overly influential studies, however the studentized residuals suggested one study might be a potential outlier.^99^ Leave-one-out analysis of the outlier still identified increased IFN-ɣ in IBS over control (SMD = 1.97, 95% CI = 1.166, 2.78, *p* < 0.0001).

Peripheral IL-1β was substantially elevated in IBS over controls (n = 6 studies,^40^^,^^53^^,^^73^^,^^76^^,^^111^^,^^123^ n = 280 controls, n = 397 IBS, SMD = 1.85, 95% CI = −0.01, 3.71, *p* = 0.051) with considerable heterogeneity (I^2^ = 98.7%) (Fig. 2d). Total participant number (R^2^ = 34.14%), IBS diagnostic criteria (R^2^ = 0%), percentage of females with IBS (R^2^ = 0%) or geographical region (R^2^ = 32.77%) did not significantly explain the observed heterogeneity. No included studies were identified as likely overly influential. There were no significant differences in peripheral 5-HT, albumin, zonulin, CtdB, CRP, IL-10, IL-8, leptin, cortisol, vinculin or norepinephrine between IBS and healthy or asymptomatic controls.

### Faecal calprotectin and valerate differentiate IBS patients from healthy/asymptomatic controls

**Fig. 3:**
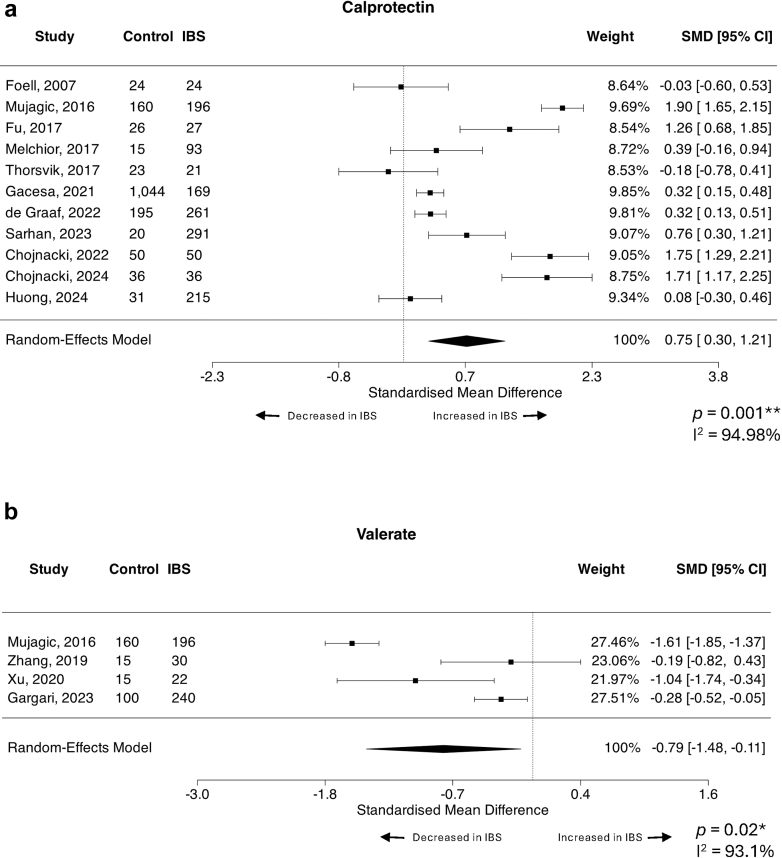
Forest plots of faecal markers that differentiate IBS patients from healthy or asymptomatic controls. Standardised mean difference (SMD) with 95% confidence intervals were assessed using a random effects model for studies reporting quantitative faecal levels of (a) calprotectin (n = 11 studies, n = 1624 controls, n = 1383 IBS) and (b) valerate (n = 4 studies, n = 290 controls, n = 488 IBS) between IBS subjects and healthy or asymptomatic control patients. Heterogeneity of the included publications was tested using the I^2^ statistic. Forest plots presented as IBS over control. A positive SMD means the assessed factor is increased in IBS over control.

Calprotectin, chromogranin A and the short chain fatty acids (SCFA) butyrate, valerate, propionate and acetate were able to be assessed by meta-analysis. There was no significant difference between IBS and healthy/asymptomatic controls regarding faecal levels of chromogranin A, butyrate, propionate or acetate (Table 2). However, calprotectin was significantly increased in IBS compared to controls across 11 studies^46^^,^^59^^,^^76^^,^^82^^,^^100^^,^^112^, ^113^, ^114^^,^^122^^,^^127^^,^^128^ including 1624 controls and 1383 IBS (SMD = 0.75, 95% CI = 0.30, 1.21, *p* = 0.001) with considerable heterogeneity (I^2^ = 94.98%) (Fig. 3a).This heterogeneity was not explained by total number of participants, IBS diagnostic criteria, percentage of female patients with IBS or geographical region (R^2^ = 0% for all). No studies were identified as likely overly influential, and the Egger's test did not suggest potential funnel plot asymmetry (*p* = 0.79) (Supplementary Figure S2C).

**Table 2:** IBS-diarrhoea versus control, all possible datasets meta-analysed.

Factor	Blood or faecal	Studies (n)	Control (n)	IBS-D (n)	Average SMD (95% CI)	*p* value	I^2^ statistic	Study references
Serotonin	Blood	3	239	122	4.13 (−4.45, 12.72)	0.345	99.89%	Yu, 2016^37^; Thijssen, 2016^48^; James, 2023^98^
Albumin	Blood	3	248	219	−0.39 (−0.68, −0.11)	0.007∗∗	45.08%	Yao, 2023^39^; Borecki, 2021^133^; Huang, 2024^101^
Anti-cytolethal distending toxin B	Blood	4	376	25331	0.33 (−0.26, 0.92)	0.273	90.89%	Pimentel, 2015^69^; Talley, 2019^51^; Hanevik, 2022^105^; Barros, 2025^24^
Butyrate	Faeces	3	44	73	−0.26 (−0.63, 0.12)	0.184	0.00%	Tian, 2019^45^; Zhang, 2019^35^; Xu, 2020^40^
IL-6	Blood	4	169	153	2.53 (0.86, 4.21)	0.003∗∗	96.33%	Rana, 2012^66^; Seyedmirzaee, 2016^55^; Russo, 2018^62^; Xu, 2020^40^
IL-8	Blood	3	107	90	3.22 (−0.51, 6.95)	0.091	98.52%	Seyedmirzaee, 2016^55^; Russo, 2018^62^; Xu, 2020^40^
IL-10	Blood	5	257	251	−1.01 (−2.97, 0.96)	0.315	98.79%	Gao, 2013^111^; Rana, 2012^66^; Zhu, 2019^32^; Xu, 2020^40^; Bilooka, 2021^135^
TNF-⍺	Blood	4	175	149	7.93 (−4.02, 19.88)	0.194	99.93%	Rana, 2012^66^; Seyedmirzaee, 2016^55^; Russo, 2018^62^; Bilooka, 2021^135^
Anti-vinculin antibodies	Blood	3	555	2517	0.22 (−0.24, 0.68)	0.341	91.11%	Pimentel, 2015^69^; Talley, 2019^51^; Barros, 2024^24^
Zonulin	Blood	4	310	188	0.18 (−0.21, 0.56)	0.373	69.26%	Singh, 2019^54^; Barbaro, 2020^138^; Talley, 2020^50^; Rezazadegan, 2022^63^

Positive SMD means factor is increased in IBS-D over control, negative SMD means factor is decreased in IBS-D over control. ∗∗*p* < 0.01.

Faecal valerate was the only SCFA that differed between IBS and controls, with levels significantly reduced in IBS across four studies,^30^^,^^35^^,^^40^^,^^76^ accounting for 290 controls and 488 IBS subjects (SMD = −0.79, 95% CI = −1.48, −0.11, *p* = 0.02) with substantial heterogeneity (I^2^ = 93.1%) (Fig. 3b). Diagnostic criteria explained heterogeneity across studies (QM = 51.22, *p* < 0.0001, R^2^ = 99.13%, I^2^ = 5.22%). The use of the Rome IV criteria resulted in significantly higher effect sizes compared to Rome III (β = 1.27, 95% CI 0.92, 1.61, *p* < 0.0001). Total participants (R^2^ = 0%), percentage of females (R^2^ = 2.72%) and geographical region (R^2^ = 0%) did not contribute to heterogeneity. While Cook's distances did not identify any overly influential studies, the studentized residuals suggested one study^76^ may be a potential outlier in the random effects model. Valerate was still decreased in IBS compared to control following the removal of the outlying study (SMD = −0.43, 95% CI = −0.86, −0.01, *p* = 0.046).

### Albumin is reduced, while IL-6 is increased in IBS-D patients compared to healthy/asymptomatic controls

**Fig. 4:**
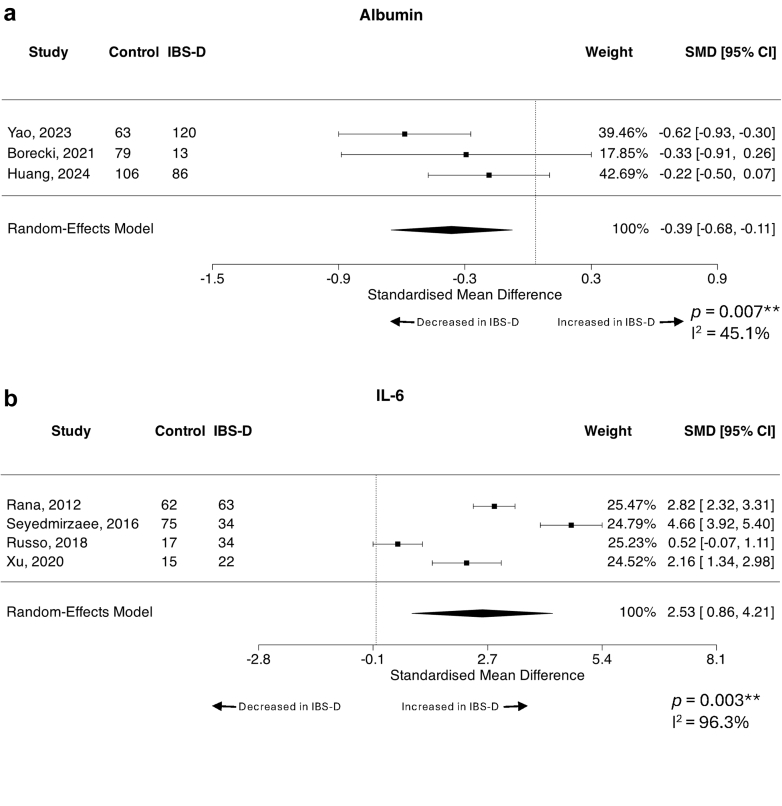
Forest plots of peripheral markers that differentiate IBS-D patients from healthy or asymptomatic control subjects. Standardised mean difference (SMD) with 95% confidence intervals were assessed using a random effects model for studies reporting quantitative systemic levels of (a) albumin (n = 3 studies, n = 248 controls, n = 219 IBS-D) and (b) IL-6 (n = 4 studies, n = 169 controls, n = 153 IBS-D) between IBS-D subjects and healthy or asymptomatic control patients. Heterogeneity of the included publications was tested using the I^2^ statistic. Forest plots presented as IBS-D over control where a positive SMD means the assessed factor is increased in IBS-D over control.

Given the predominance of subjects with IBS-D across included studies, we next assessed factors that may differentiate IBS-D from healthy or asymptomatic controls, including peripheral 5-HT, albumin, anti-CtdB, IL-6, IL-8, IL-10, TNF-⍺, zonulin, anti-vinculin and faecal butyrate. While the majority of these factors were unchanged between IBS-D and controls, we did observe a significant decrease in the levels of peripheral albumin in patients with IBS-D compared to controls across three included studies^39^^,^^101^^,^^133^ assessing 248 controls and 219 subjects with IBS-D (SMD = −0.39, 95% CI = −0.68, −0.11, *p* = 0.007) (Fig. 4a). The included studies demonstrated moderate heterogeneity (I^2^ = 45.1%) and none of the studies were identified as potential outliers or overly influential. All studies analysed used the Rome IV criteria and were conducted in Asia, and neither total participant number nor percentage of females with IBS explained the heterogeneity observed (0% for both).

In contrast, patients with IBS-D had increased peripheral IL-6 levels compared to controls when four studies^40^^,^^55^^,^^62^^,^^66^ were analysed, representing a total of 153 IBS-D and 169 controls (SMD = 2.53, 95% CI = 0.86, 4.21, *p* = 0.003), with considerable heterogeneity (96.3%) (Fig. 4b). Total participant number (R^2^ = 18.65%), IBS diagnostic criteria (0%), percentage of females with IBS (0%) and geographical region (46.69%) did not significantly explain the observed heterogeneity. None of the studies were identified as potential outliers or overly influential. We initially aimed to analyse IBS-C compared to IBS-D to determine if there were subtype specific differences however, this was only possible for peripheral 5-HT and CRP, which were both unchanged between subtypes (Table 3).

**Table 3:** IBS-constipation versus IBS-diarrhoea, all possible datasets meta-analysed.

Factor	Blood or faecal	Studies (n)	IBS-C (n)	IBS-D (n)	Average SMD (95% CI)	*p* value	I^2^ statistic	Study references
Serotonin	Blood	3	89	128	0.02 (−0.25, 0.30)	0.86	0.00%	James, 2023^98^; Chojnacki, 2024^127^; Thijssen, 2016^48^
C-reactive protein	Blood	3	101	60	−1.49 (−3.49, 0.52)	0.145	96.08%	Bilooka, 2021^135^; Borecki, 2021^133^; Tuncel, 2025^28^

Positive SMD means factor is increased in IBS-C over IBS-D, negative SMD means factor is increased in IBS-D over IBS-C.

### IBS patients have increased peripheral albumin levels compared to patients with organic gastrointestinal conditions

**Fig. 5:**
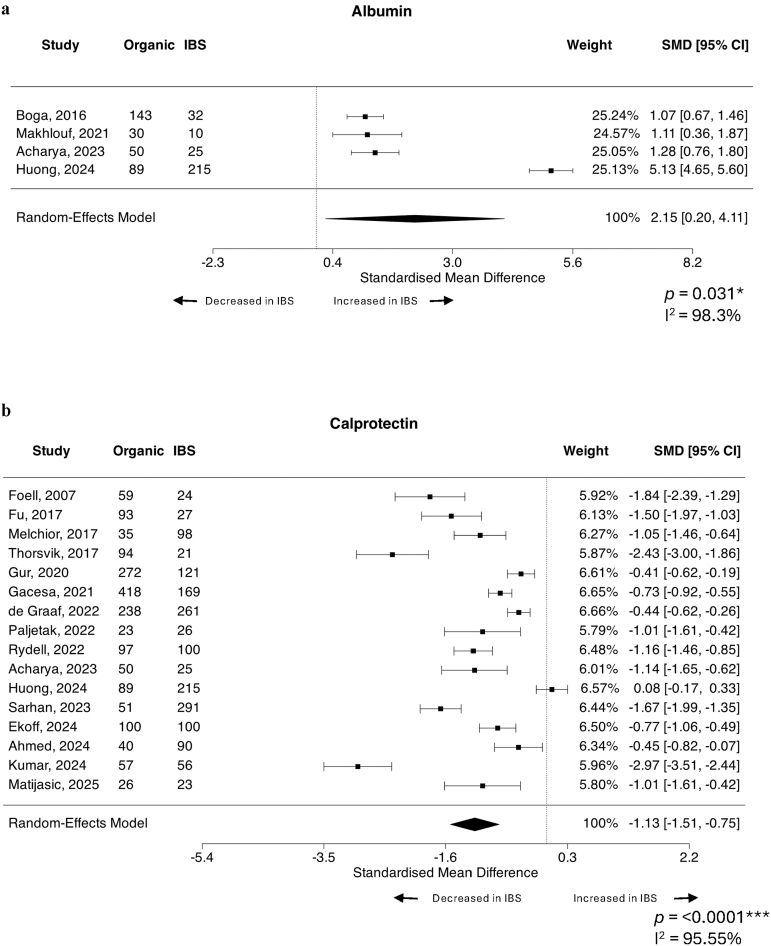
Forest plots of peripheral and faecal markers that differentiate IBS patients from patients with other organic gastrointestinal diseases. Standardised mean difference (SMD) with 95% confidence intervals were assessed using a random effects model for studies reporting quantitative systemic levels of (a) albumin (n = 4 studies, n = 312 organic gastrointestinal disease, n = 282 IBS) and (b) faecal levels of calprotectin (n = 16 studies, n = 1685 organic gastrointestinal disease, n = 1591 IBS) between IBS subjects and patients with organic gastrointestinal diseases, the majority of which are inflammatory bowel diseases. Heterogeneity of the included publications was tested using the I^2^ statistic. Forest plots presented as IBS over organic disease where a positive SMD means factor is increased in IBS over organic disease, negative SMD means factor is decreased in IBS compared to organic disease.

Finally, we assessed all factors suitable for meta-analysis between patients with IBS and other organic GI conditions (predominantly IBD). Suitable factors included albumin, anti-CtdB, anti-vinculin, CRP, IL-6 and zonulin in the periphery, and calprotectin in faeces (Table 4). Regarding peripheral factors, the only significant difference observed was an increase in albumin levels in IBS compared to controls, with four studies included,^85^^,^^100^^,^^134^^,^^144^ assessing a total of 282 IBS and 312 organic disease subjects (SMD = 2.15, 95% CI = 0.20, 4.11, *p* = 0.031) with considerable heterogeneity (I^2^ = 98.3%) (Fig. 5a). Total participant number explained some heterogeneity across studies (R^2^ = 62.4% I^2^ = 95.64%, QM = 5.77, *p* = 0.02), with larger sample sizes associated with higher effect sizes (β = 0.01, 95% CI 0.0027, 0.0265). The percentage of females with IBS did not substantially contribute (R^2^ = 0%) and all included studies were conducted in Asia. While no studies were identified as overly influential, the studentized residuals did suggest that one study^100^ may be an outlier within the model. However, leave-one-out analysis still identified increased albumin levels in IBS over organic disease (SMD = 1.14, 95% CI = 0.85, 1.43, *p* < 0.0001).

**Table 4:** IBS versus organic disease, all possible datasets meta-analysed.

Factor	Blood or faecal	Studies (n)	Organic disease (n)	IBS (n)	Average SMD (95% CI)	*p* value	I^2^ statistic	Study references
Albumin	Blood	4	312	282	2.15 (0.20, 4.11)	0.031∗	98.28%	Boga, 2016^134^; Makhlouf, 2021^85^; Archarya, 2023^144^; Huong, 2024^100^
Anti-cytolethal distending toxin B	Blood	4	458	2770	0.40 (−0.02, 0.82)	0.064	90.46%	Pimentel, 2015^69^; Talley, 2019^51^; Hanevik, 2022^105^; Barros, 2024^24^
C-reactive protein	Blood	9	649	796	−1.79 (−3.95, 0.36)	0.103	99.58%	Boga, 2016^134^; Manolakis, 2017^84^; Bilooka, 2021^135^; Makhlouf, 2021^85^; Paljetak, 2022^71^; Rydell, 2022^61^; Sarhan, 2023^59^; Huong, 2024^100^; Matijasic, 2025^23^
IL-6	Blood	4	151	286	−0.17 (−1.42, 1.08)	0.791	95.85%	Pellissier, 2014^70^; Xu, 2020^40^; Huong, 2024^100^; Matijasic, 2025^23^
Zonulin	Blood	4	200	323	−0.23 (−0.56, 0.09)	0.163	64.60%	Singh, 2019^54^; Barbaro, 2020^138^; Talley, 2020^50^; de Faria, 2021^121^
Anti-vinculin antibodies	Blood	3	429	2736	0.05 (−0.32, 0.42)	0.794	87.57%	Pimentel, 2015^69^; Talley, 2019^51^; Barros, 2024^24^
Calprotectin	Faeces	16	1685	1591	−1.13 (−1.51, −0.75)	<0.0001∗∗∗	95.55%	Foell, 2007^114^; Fu, 2017^113^; Melchior, 2017^82^; Thorsvik, 2017^46^; Gur, 2020^108^; Gacesa, 2021^112^; de Graaf, 2022^122^; Paljetak, 2022^71^; Rydell, 2022^61^; Archarya, 2023^144^; Huong, 2024^100^; Sarhan, 2023^59^; Ekoff, 2024^117^; Ahmed, 2024^143^; Kumar, 2024^29^; Matijasic, 2025^23^

Positive SMD means factor is increased in IBS over organic disease, negative SMD means factor is increased in organic disease over IBS. ∗*p* < 0.05; ∗∗∗*p* < 0.001.

As expected, faecal calprotectin was significantly higher in patients with organic GI diseases compared to IBS, analysed in 16 studies^23^^,^^29^^,^^46^^,^^59^^,^^61^^,^^71^^,^^82^^,^^100^^,^^108^^,^^112^, ^113^, ^114^^,^^117^^,^^122^^,^^143^^,^^144^ with a total of 1591 IBS and 1685 organic disease subjects (SMD = −1.013, 95% CI = −1.351, −0.75, *p* < 0.0001) with considerable heterogeneity (I^2^ = 95.6%) (Fig. 5b). However total participant number (R^2^ = 15.36%), IBS diagnostic criteria, percentage of females in cohort or geographical region (R^2^ = 0.00% for all) were not significant moderators of this heterogeneity. There was no suggestion of outliers or overly influential studies in this model, however the Egger's test did suggest funnel plot asymmetry (*p* = 0.002) (Supplementary Figure S3). The trim-and-fill method suggested no statistically significant evidence that the asymmetry was solely due to publication bias (estimate: 1 study missing on right, *p* = 0.25).

## Discussion

While IBS has conventionally been considered a “functional” condition with no overt structural or biochemical signature, recent work has highlighted abnormalities in the composition of the microbiome, host immunity and the enteric nervous system that suggest an underlying defect in microbiome-immune homoeostasis in at least some subtypes of IBS.^3^ In this review we aimed to identify serological and faecal biomarkers that could differentiate IBS and IBS-subtypes from health individuals or those with organic disease by meta-analysis. We identified 115 studies encompassing 14,169 patients that measured faecal and/or blood biomarker differences between patients with IBS and controls, and found that pyrogenic cytokines TNF-⍺, IL-6 and IFN-γ were consistently and significantly increased in the peripheral blood of IBS patients over controls, while faecal calprotectin and valerate also differentiated patients with IBS from control groups.

Increase in epithelial permeability is a hallmark of IBS with increased sugar absorption linked to symptom severity.^145^ Epithelial permeability increases the potential translocation of harmful luminal contents, including bacteria, and thus elevated pyrogenic cytokines in patients with IBS may be a result of increased bacterial exposure to the mucosal immune system. Indeed, a recent systematic review of cytokines associated with epithelial permeability identified TNF-⍺, IFN-γ and IL-1β as the most frequently identified causative cytokines.^146^ In our meta-analysis these three cytokines were elevated in IBS overall versus controls, although in the case of IL-1β, this did not reach significance. Epithelial tight junction dysfunctional has been proposed as a mechanism for this permeability with both increased serum zonulin^54^ and reduced mucosal e-cadherin^147^ expression associated in particular with IBS-D. In our identified studies, IBS-D was the most common subtype of IBS reflecting over half of the identified cases, nevertheless only IL-6 discriminated IBS-D from health controls, although this was likely due to the comparatively fewer number of studies that specifically compared IBS-D subgroups in the identified studies. Given the previous studies that suggest permeability is linked to the IBS-D subtype^54^^,^^147^ and the association between permeability and increased cytokines, it is possible that IBS-D may represent a more immune-involved IBS subtype than IBS-C, although the heterogeneity and lack of sub-typing in many of our identified studies make this difficult to determine. Patients with IBS-D had significantly lower serum albumin levels when compared healthy groups. Low serum albumin is a non-specific marker of malnutrition^148^ where prolonged diarrhoea causes protein and nutrient malabsorption^149^ and this can lead to lower albumin production by the liver. Hypoalbuminemia has been assessed as a marker of malnutrition and inflammation in IBD^150^ and serum albumin was found to be significantly lower in organic disease when compared to IBS-D, likely reflective of the greater severity of disease in these conditions.

Faecal calprotectin (fCal) is a neutrophil derived protein stool biomarker that is routinely used to discriminate IBD inflammation from IBS in patients with unexplained abdominal pain and diarrhoea.^151^ In clinical practice, “normal” fCal levels are ascribed to both healthy controls and IBS, but some studies suggest abnormally high fCal levels in IBS.^82^ A prospective study of 93 patients with IBS by Melchior et al. found that over one third of patients had elevated fCal with 17% over 100 μg/g.^82^ Our meta-analysis supported this finding showing that although fCal levels distinguish IBS and organic disease, the majority of which were IBD, they are still significantly higher in IBS over healthy controls. In our analyses, average faecal calprotectin was 58.56 μg/g for IBS patients compared to 468.51 μg/g for IBD (across 14 studies), while the average fCal concentration was 25.48 μg/g in healthy controls from 11 studies. These values are slightly higher than those proposed to discriminate IBS from IBD by Dhaliwal et al.^152^ which suggests a cut off of 50 μg/g is sufficient, but similar to the cut offs proposed by D'Haens et al.^153^ who found a median of 54 μg/g of fCal in patients with IBS, although fCal cutoffs are still subject to variations based factors such as analysis methods and geographical location. The elevated fCal may again be linked to active inflammation in patients with IBS, although none of these studies linked fCal levels to a specific IBS subtype.^82^ Patients with IBD in remission often report IBS-like symptoms and these symptoms are associated with elevated TNF-⍺ and intestinal permeability.^154^ It therefore seems likely that, in at least some patients with IBS, immune mediated permeability is an underlying cause of symptoms, however in the organic disease cases, this is more severe and consistent overall. Faecal valerate, a short chain fatty acid (SCFA) was also found to distinguish IBS and health individuals with patients with IBS exhibiting lower faecal valerate levels. Interestingly recent work by Gargari et al.^30^ stratified non-constipation patients into those with low or high carbotypes based on their SCFA profiles and showed that although valerate levels were reduced in the IBS population overall, the carbtoype with lower valerate levels had lower pain symptoms. Thus while specific SCFA markers may serve as biomarkers, it is likely important that SCFA levels overall are more informative in discerning IBS subtypes.

There were some limitations to our study that should be considered in context. Firstly, there was a high degree of heterogeneity in the study analyses and subtyping and this is likely reflected the in high heterogeneity across all of the biomarkers studied. Unfortunately, many studies in the field fail to report IBS subtypes or under-represent specific subtypes, (such as IBS-C as seen in this study) in recruitment. This is reflected in our analysis where there were few studies with data for IBS-C to allow meta-analysis. Further, given the predominance of IBS-D in the studies suitable for meta-analysis, some of the markers identified in this study may not be applicable to IBS-C or IBS-M and require larger, subtype specific studies to validate. Our study identified total participant number as a significant moderator of heterogeneity across several factors assessed, with larger studies tending to show greater effect sizes. This supports the hypothesis that many studies investigating peripheral factors in IBS may be underpowered, potentially contributing to inconsistent findings across the broader IBS literature. While our study identified key markers related to inflammation, it is notable that there were few studies that assessed gut-brain markers and hormones such as leptin and cortisol and these showed no differences in our study, although this may be related to the power. Similarly, there were few studies that identified post-infectious (PI) versus non-PI IBS which may have limited identifying markers associated with infection, such as anti- CdtB and vinculin. This may be an important confounder to identifying biomarkers as the prevalence of post-infectious IBS is approximately 10% of cases and more likely to manifest as IBS-M and IBS-D.^155^ These cases may have different biomarker profiles to IBS that is considered idiopathic.

In summary, we have performed a comprehensive analysis of blood and faecal biomarkers in IBS and identified consistent cytokine and stool biomarkers associated with IBS. These biomarkers may reflect increased epithelial permeability and thus low-grade bacteraemia in patients with IBS and this may be suggestive of an underlying cause of IBS symptoms currently overlooked. In addition, care should be taken when using albumin and fCal to distinguish organic GI diseases from IBS-D. While caution should be taken in suggesting any one marker can be used to identify IBS, these data do add further evidence to the view that IBS pathology has an underlying immune basis.

## Contributors

The study was conceptualised by SK, GLB and the methodology developed by GLB, SF, KD, MJ, NJT. The search strategy was developed and completed by FR and GLB, with investigation conducted by GLB, FR, JAW, SK. The original draft was written by GLB and SK, and all authors read and approved the final version of the manuscript.

## Data sharing statement

All data contained in this manuscript is sourced from previously published works cited in the reference list. All data relevant to the search strategy and meta-analysis process are available in the main text or the Supplementary Materials, further information is available from the authors upon reasonable request to the corresponding author. All coding packages and R workflows are publicly available but we have also included the code used produce the meta-analysis and associated plots included in the Supplementary.

## Declaration of interests

GLB: Patent: “Diagnostic marker for functional gastrointestinal disorders” (Australian Patent Application WO2022256861A1) via the University of Newcastle and UniQuest (University of Queensland).

FR: Nothing to disclose.

JAW: Nothing to disclose.

MJ: Funding from NHMRC and Nanosonics, consulting fees from Nanosonics

KD: Nothing to disclose.

SF: Nothing to disclose.

NJT: Disclosures: Brown University & Agency for Health Care Research and Quality (fibre and laxation), Rome Foundation (member gastroduodenal committee), Biocodex (FD diagnostic tool), Microba (microbiome), Comvita Manuka Honey (FD trial consulting), BluMaiden (microbiome) outside the submitted work. In addition, Dr. Talley has Licencing Questionnaires: Nepean Dyspepsia Index (NDI) 1998 licenced to MAPI, Talley Bowel Disease Questionnaires licenced to Mayo Clinic. Patents: “Diagnostic marker for functional gastrointestinal disorders” Australian Provisional Patent Application 2021901692, ”Methods and compositions for treating age-related neurodegenerative disease associated with dysbiosis” US Patent Application No. 63/537,725, “Compositions and methods for the treatment of oesophageal disorders” Australian Provisional Patent ID 2025902002. Boards: Asia Pacific Association of Medical Editors (APAME) Board (President 2023–2026), Doctors for the Environment (Chair 2024-present), Gastroenterological Society of Australia (GESA) Board (2025–2027). Editorial: UptoDate (Section Editor), Mayo Clinic Proceeding Associate Editor (2023-present). Financial support: Dr. Talley is supported by funding from the National Health and Medical Research Council (NHMRC) to the Centre for Research Excellence in Digestive Health and he holds an NHMRC Investigator grant.

SK: Patent: “Diagnostic marker for functional gastrointestinal disorders” (Australian Patent Application WO2022256861A1) via the University of Newcastle and UniQuest (University of Queensland). Grants from National Health and Medical Research Council (Ideas Grant and Centre for Research Excellence), grants from Viscera Labs (Research contract), grants from Microba Life Science (Research contract), personal fees from Gossamer Bio, personal fees from Anatara Lifescience, personal fees from Immuron, personal fees from Microba Life Science.
