# Supplementary material for: Serological and faecal markers of irritable bowel syndrome: a systematic review and meta-analysis
Source: eBioMedicine. 2026 Mar 6;126:106198. doi: 10.1016/j.ebiom.2026.106198 (PMC12992513; doi:10.1016/j.ebiom.2026.106198)
Supplement: Supplementary Material [file mmc1.pdf]

Supplementary Table 1: PRISMA guidelines 2020 checklist

Section and Topic	Item #	Checklist item	Location where item is reported
**TITLE**			
Title	1	Identify the report as a systematic review.	Title page (pg 1)
**ABSTRACT**			
Abstract	2	See the PRISMA 2020 for Abstracts checklist.	
**INTRODUCTION**			
Rationale	3	Describe the rationale for the review in the context of existing knowledge.	Introduction (pg 6-7 Lines 35-38)
Objectives	4	Provide an explicit statement of the objective(s) or question(s) the review addresses.	Introduction (pg 6, Lines 39-42)
**METHODS**			
Eligibility criteria	5	Specify the inclusion and exclusion criteria for the review and how studies were grouped for the syntheses.	Supplementary Table 3, Methods lines 61-66. Results Lines 125-127
Information sources	6	Specify all databases, registers, websites, organisations, reference lists and other sources searched or consulted to identify studies. Specify the date when each source was last searched or consulted.	Methodology Lines 47-49
Search strategy	7	Present the full search strategies for all databases, registers and websites, including any filters and limits used.	Methodology Lines 51-54, Supplementary Table 2
Selection process	8	Specify the methods used to decide whether a study met the inclusion criteria of the review, including how many reviewers screened each record and each report retrieved, whether they worked independently, and if applicable, details of automation tools used in the process.	Methodology Lines 59-68
Data collection process	9	Specify the methods used to collect data from reports, including how many reviewers collected data from each report, whether they worked independently, any processes for obtaining or confirming data from study investigators, and if applicable, details of automation tools used in the process.	Methodology Lines 71-84
Data items	10a	List and define all outcomes for which data were sought. Specify whether all results that were compatible with each outcome domain in each study were sought (e.g. for all measures, time points, analyses), and if not, the methods used to decide which results to collect.	Methodology Lines 86-93
	10b	List and define all other variables for which data were sought (e.g. participant and intervention characteristics, funding sources). Describe any assumptions made about any missing or unclear information.	Methodology Lines 86-124
Study risk of bias assessment	11	Specify the methods used to assess risk of bias in the included studies, including details of the tool(s) used, how many reviewers assessed each study and whether they worked independently, and if applicable, details of automation tools used in the process.	Methodology Lines 115-120
Effect measures	12	Specify for each outcome the effect measure(s) (e.g. risk ratio, mean difference) used in the	Methodology Lines 97-101

Section and Topic	Item #	Checklist item	Location where item is reported
		synthesis or presentation of results.	
Synthesis methods	13a	Describe the processes used to decide which studies were eligible for each synthesis (e.g. tabulating the study intervention characteristics and comparing against the planned groups for each synthesis (item #5)).	Methodology Lines 95-102
	13b	Describe any methods required to prepare the data for presentation or synthesis, such as handling of missing summary statistics, or data conversions.	Methodology Lines 86-93<br>Methodology Lines 76-83;
	13c	Describe any methods used to tabulate or visually display results of individual studies and syntheses.	Methodology Lines 95-97
	13d	Describe any methods used to synthesize results and provide a rationale for the choice(s). If meta-analysis was performed, describe the model(s), method(s) to identify the presence and extent of statistical heterogeneity, and software package(s) used.	Methodology Lines 85-124
	13e	Describe any methods used to explore possible causes of heterogeneity among study results (e.g. subgroup analysis, meta-regression).	Methodology Lines 105-113
	13f	Describe any sensitivity analyses conducted to assess robustness of the synthesized results.	Methodology Lines 113-115
Reporting bias assessment	14	Describe any methods used to assess risk of bias due to missing results in a synthesis (arising from reporting biases).	
Certainty assessment	15	Describe any methods used to assess certainty (or confidence) in the body of evidence for an outcome.	Methodology Lines 113-115
**RESULTS**			
Study selection	16a	Describe the results of the search and selection process, from the number of records identified in the search to the number of studies included in the review, ideally using a flow diagram.	Figure 1
	16b	Cite studies that might appear to meet the inclusion criteria, but which were excluded, and explain why they were excluded.	Figure 1
Study characteristics	17	Cite each included study and present its characteristics.	Supplementary Table 4
Risk of bias in studies	18	Present assessments of risk of bias for each included study.	
Results of individual studies	19	For all outcomes, present, for each study: (a) summary statistics for each group (where appropriate) and (b) an effect estimate and its precision (e.g. confidence/credible interval), ideally using structured tables or plots.	All figures, tables, supplementary figures and tables;<br>Results pg12-18
Results of syntheses	20a	For each synthesis, briefly summarise the characteristics and risk of bias among contributing studies.	All figures, tables, supplementary figures and tables;<br>Results pg12-18
	20b	Present results of all statistical syntheses conducted. If meta-analysis was done, present for each the summary estimate and its precision (e.g. confidence/credible interval) and measures of statistical heterogeneity. If comparing groups, describe the direction of the effect.	Tables 1-4<br>Figures 2-5

Section and Topic	Item #	Checklist item	Location where item is reported
	20c	Present results of all investigations of possible causes of heterogeneity among study results.	Results pg12-18
	20d	Present results of all sensitivity analyses conducted to assess the robustness of the synthesized results.	Results pg12-18
Reporting biases	21	Present assessments of risk of bias due to missing results (arising from reporting biases) for each synthesis assessed.	
Certainty of evidence	22	Present assessments of certainty (or confidence) in the body of evidence for each outcome assessed.	Results pg12-18
**DISCUSSION**			
Discussion	23a	Provide a general interpretation of the results in the context of other evidence.	Pg 19-22
	23b	Discuss any limitations of the evidence included in the review.	Pg 19-22, Lines 368-380
	23c	Discuss any limitations of the review processes used.	Pg 19-22, Lines 360-368
	23d	Discuss implications of the results for practice, policy, and future research.	Pg 19-22, Lines 382-389
**OTHER INFORMATION**			
Registration and protocol	24a	Provide registration information for the review, including register name and registration number, or state that the review was not registered.	Prospero registration of review was done by the first author, registration details on pg 8
	24b	Indicate where the review protocol can be accessed, or state that a protocol was not prepared.	Methodology of manuscript (pg 8-11)
	24c	Describe and explain any amendments to information provided at registration or in the protocol.	NA
Support	25	Describe sources of financial or non-financial support for the review, and the role of the funders or sponsors in the review.	Pg 3
Competing interests	26	Declare any competing interests of review authors.	Pg 23-24
Availability of data, code and other materials	27	Report which of the following are publicly available and where they can be found: template data collection forms; data extracted from included studies; data used for all analyses; analytic code; any other materials used in the review.	Extracted detailed data can be obtained from the authors upon reasonable request, all data relevant to analyses included is available within manuscript and/or supplementary files

Supplementary Table 2: Systematic search strategy

Line	Term or Logic
1	Irritable bowel syndrome
2	IBS
3	irritable colon
4	disorder of gut-brain interaction
5	DGBI
6	functional gastrointestinal disorder
7	FGID
8	1 or 2 or 3 or 4 or 5 or 6 or 7
9	Biomarker* or Biomarkers
10	Plasma
11	peripheral blood mononuclear cell or leukocytes, monocytes
12	blood or Blood
13	serum
14	PBMC
15	sera*
16	marker
17	factor
18	protein or Proteins
19	hormone or Hormones
20	cytokine or Cytokines
21	immun*
22	biochem*
23	9 or 10 or 11 or 12 or 13 or 14 or 15 or 16 or 17 or 18 or 19 or 20 or 21 or 22
24	8 and 23
25	Limit 24 to (humans and yr="1992-current" and "all adult"

Supplementary Table 3: Search inclusion and exclusion criteria

**Inclusion criteria**	**Exclusion criteria**
Adults (≥18 years old) with IBS diagnosed by a recognised criteria with healthy or outpatient controls and/or organic gastrointestinal conditions (eg. IBD).	Pediatric studies
Manuscripts with an identified control population and standard diagnostic approaches	Reviews, theses and case studies
Papers published from 1992 until now (introduction of the Manning criteria and Rome criterias)	Animal or ex vivo studies
Quantification of blood and faecal markers data included in manuscript	
Studies written in English with a full text available	

Supplementary Table 4: Overview of all studies selected as suitable for inclusion in systematic review

	Study info							IBS cohort						Healthy/asymptomatic control cohort					Organic disease control cohort				
Number	First author	Year	Journal	Country	Study design	Blood factors assessed ?	Faecal factors assessed?	Diagnostic criteria	Sample size	Female (n)	Age	Age measure	Subtype	Sample size	Female (n)	Age	Age measure	Subtype	Sample size	Female (n)	Age	Age measure	Subtype
1	Gorard	1995	Scandinavian Journal of Gastroenterology	United Kingdom	Case control	Y	N	Rome I	14	10	30 (19-57)	Median	Not reported	16	9	26.5 (18-54)	Median	Healthy	9	6	38 (19-56)	Median	6 UC<br>3 CD
2	Bearcroft	1998	Gut	United Kingdom	Case control	Y	N	Manning criteria	5	5	33 (18-54)	Median	5 IBS-D	6	1	26 (18-32)	Median	Healthy	0	0	0	0	0
3	Qasim	2003	Gut	Ireland	Case control	Y	N	Rome II	20	18	29 (20-62)	Mean	18 IBS-D<br>2 unspecified	15	11	28 (22-49)	Mean	Healthy	0	0	0	0	0
4	Kilkens	2004	Lipids	Netherlands	Case control	Y	N	Rome II	23	14	32.9±2.3	semn	23 IBS-D	23	14	28.6±3.3	Mean±SEM	Healthy	0	0	0	0	0
5	Ohman	2005	Clinical Gastroenterology and Hepatology	Sweden	Case control	Y	N	Rome II	33	19	42±12	Mean	20 IBS-D<br>4 IBS-C<br>9 IBS-M	15	7	53±8	Mean	Healthy	23	10	42±11	Mean	UC
6	Yazar	2005	Scottish Medical Journal	Turkey	Case control	Y	N	Rome I	41	14<br>19	40.1±1.9<br>37.3±2.1	Mean	19 IBS-D<br>22 IBS-C	18	13	36.3±2.1	Mean	Healthy	0	0	0	0	0
7	Lettesjo	2006	Scandinavian Journal of Gastroenterology	Sweden	Case control	N	Y	Rome II	46	33	45 (18-71)	Median	19 IBS-D<br>16 IBS-C<br>11 IBS-M	20	13	35 (20-53)	Median	Healthy	18	17	61 (26-78)	Median	Collagenous colitis
8	Dai	2007	Scandinavian Journal of Gastroenterology	China	Case control	N	Y	Rome II	25	10	52.4 (16-68)	Mean	Not reported	34	16	42 (20-62)	Mean	Healthy	55	18<br>3	45.69 (14-68)<br>31.46 (19-43)	Mean	42 UC<br>13 CD
9	Foell	2007	Gastroenterology	Germany	Case control	Y	Y	Rome II	24	21	46 (16-70)	Median	Not reported	24	10	35 (17-43)	Median	Healthy	59	21<br>11	34 (19-62)<br>46 (22-71)	Median	32 CD<br>27 UC

10	Houghton	2007	Neurogastroenterology and Motility	United Kingdom	Case control	Y	N	Rome II	35	13IBS-D<br>14 IBS-C	30 (19-42) IBS-D<br>30 (23-53) IBS-C	Mean	18 IBS-D<br>17 IBS-C	16	10	23 (18-39)	Mean	Healthy	0	0	0	0	0
11	Zuo	2007	Clinical and Experimental Allergy	China	Case control	Y	N	Rome II	37	25	36	Mean	Not reported	20	14	36.5	Mean	Healthy	0	0	0	0	0
12	Otten	2008	Clinical Chemistry and Laboratory Medicine	Netherlands	Case control	N	Y	Not reported	91	49	52.3	Mean	Not reported	0	0	0	NA	NA	23	12	44.5	Mean	6 CD<br>5 UC<br>12 unspecified colitis
13	Christmas	2010	Clinical Chemistry and Laboratory Medicine	United Kingdom (American control group)	Case control	Y	N	Manning criteria	8	4	40±15	Mean	8 IBS-D	8	4	36.3±4	Mean	Healthy	0	0	0	NA	0
14	Hattori	2010	Neurogastroenterology and Motility	Japan	Case control	Y	N	Rome II	12	0	21.5±0.4	Mean±SEM	7 IBS-D<br>3 IBS-C<br>2 IBS-M	12	0	21.3±0.6	Mean±SEM	Healthy	0	0	0	NA	0
15	Carroccio	2011	Clinical Gastroenterology and Hepatology	Italy	Case control	N	Y	Rome II	160	127	33 (18-60)	Median	Not reported	50	38	30 (18-60)	Median	Healthy	40	30	31 (18-59)	Median	16 Coeliac disease<br>18 active CD<br>6 giardiasis
16	Jones	2011	Alimentary Pharmacology and Therapeutics	USA	Case control	Y	N	Rome III	168	48 IBS-C<br>37 IBS-D<br>43 IBS-M	38.8±12.6 IBS-C<br>41.1±13.6 IBS-D<br>37.5±13.3 IBS-M	Mean	60 IBS-C<br>57 IBS-D<br>51 IBS-M	76	60	38.8±12.4	Mean	Healthy	0	0	0	NA	NA
17	Stankiewicz	2011	Central European Journal of Immunology	Poland	Case control	Y	N	Rome III	17	15	51.8 (24-81)	Mean	3 IBS-C<br>5 IBS-D<br>9 IBS-M	20	Not reported	Not reported	Not reported	NA	NA	NA	NA	NA	NA
18	Tursi	2011	Minerva Gastroenterologica E Dietologica	Italy	Case control	N	Y	Rome II	16	11	58.2±13	Median±SD	Not reported	16	11	60.6±12	Median±SD	Healthy	16	9	36.9±11	Median±SD	Active UC
19	Chang	2012	American Journal of Gastroenterology	USA	Case control	Y	N	Rome II	45	26	43.3±2.4 M<br>37.7±2.0 F	Mean±SEM	16 IBS-C<br>15 IBS-D<br>14 IBS-M	41	22	42.7±2.5 M<br>33.0±1.9 F	Mean±SEM	Healthy	0	0	0	0	0

20	Schmulson	2012	American Journal of Gastroenterology	Mexico	Cohort	Y	N	Rome II	62	34	31.1 (28.1, 34.1)	Mean (95% CI)	15 IBS-D<br>16 IBS-C<br>31 IBS-M	116	57	34.2 (31.8, 36.6)	Mean (95% CI)		0	0	0	0	0
21	Ahmed	2013	PLoS one	United Kingdom	Case control	N	Y	Manning criteria	30	23	24 (19-65)	Mean	30 IBS-D	109	69	33 (24-76)	Mean	Healthy	110	55	39 (18-80) CD<br>38 (18-77) UC	Mean	62 CD<br>48 UC
22	Lu	2014	Disease Markers	China	Case control	Y	N	Rome III	120 FGIDs	68	39.05±15.20	Mean	FD<br>IBS	60	Not reported	Not reported	Not reported	Healthy	240	56 active<br>68 remission	34.51±12.04 active<br>36.38±13.92 remission	Mean	112 active CD<br>128 remission
23	Pellissier	2014	PLoS one	France	Case control	Y	N	Rome II	26	19	38±11	Mean	7 IBS-D<br>1 IBS-C<br>18 IBS-M	26	18	36±10	Mean	Healthy	21	11	40±11	Mean	CD in remission
24	Tooth	2014	Gut	United Kingdom	Case control	N	Y	Rome III	36	25	39±2	Mean±SEM	36 IBS-D	9	6	25.4±2.3	Mean±SEM	Healthy	0	0	0	0	0
25	Farup	2015	BMC Gastroenterology	Norway	Case control	N	Y	Rome III	25	13	46.2±12.9	Mean	8 IBS-C<br>9 IBS-D<br>7 IBS-M<br>1 IBS-U	25	15	49.2±14.6	Mean	Idiopathic depression	0	0	0	0	0
26	Zhen	2015	Molecular Medicine Reports	China	Case control	Y	N	Rome III	42	27	36.4±6.9 males<br>38.1±5.9 females	Mean	42 IBS-D	20	12	37.9±7.8 males<br>35.1±8.1	Mean	Healthy	0	0	0	0	0
27	Boga	2016	Journal of Investigative Medicine	Turkey	Case control	Y	N	Rome III	32	21	41.3±12.8	Mean	Not reported	50	25	40.3±11.8	Mean	Healthy	143	84	40.2±11.7	Mean	83 UC<br>60 CD
28	Mujagic	2016	Scientific Reports	Netherlands	Case control	Y	Y	Rome III	196	138	44.8±16.4	Mean	71 IBS-D<br>34 IBS-C<br>78 IBS-M<br>13 IBS-U	160	98	43.9±19.2	Mean	Healthy	0	0	0	0	0
29	Thijssen	2016	Alimentary Pharmacology and Therapeutics	Netherlands	Case control	Y	N	Rome III	154	108	44.5±16.3	Mean	52 IBS-D<br>33 IBS-C<br>60 IBS-M<br>9 IBS-U	137	84	44.2±19.3	Mean	Healthy	0	0	0	0	0

30	Yu	2016	World Journal of Gastroenterology	China	Case control	Y	N	Not reported	30	14	39±21	Mean	30 IBS-D	30	15	42.5±19.5	Mean	Healthy	33	15	39±17	Mean	UC remission
31	Zhang	2016	Digestive Diseases and Sciences	China	Case control	Y	Y	Rome III	12	4	35 (25-51)	Median	Not reported	44	18	39 (33-60	Median	Healthy	115	28 UC<br>12 CD	43 (22-71) UC<br>29 (15-62) CD	Median	78 UC<br>37 CD
32	Da Silva Kotze	2017	Revista da Associacao Medica Brasileira	Brazil	Cross sectional	Y	N	Not reported	40	31	52 (21-77)	Median	Not reported	0	0	0	0	0	65	22 CD<br>21 UC	39.5 (16-73) CD<br>39 (16-70) UC	Median	38 CD<br>27 UC
33	Fu	2017	Scientific Reports	China	Case control	N	Y	Rome III	27	9	34.0 (27.0-40.0)	Median	Not reported	26	11	32 (27.0-43.3)	Median	Healthy	93	16 CD<br>18 UC	28.5 (24-36.8) CD<br>39.0 (33.5-47.5) UC	Median	44 CD<br>49 UC
34	Li	2017	Clinics and Research in Hepatology and Gastroenterology	China	Case control	Y	N	Rome III	38	21	48.5±9.5	Mean	38 IBS-C	22	13	48.1±9.8	Mean	Healthy	0	0	0	0	0
35	Manolakis	2017	World Journal of Gastroenterology	Greece	Case control	Y	N	Rome III	62	26	49±19.4	Mean	40 IBS-D<br>22 IBS-M	180	66	46.7±12.3	Mean	Healthy	180	36 UC<br>39 CD	50.2±13.4 UC<br>42.3±15.6 CD	Mean	96 UC<br>84 CD
36	Melchior	2017	United European Gastroenterology Journal	France	Case control	N	Y	Rome III	93	68	41±13.9	Mean	62 IBS-D<br>14 IBS-C<br>16 IBS-M<br>1 IBS-U	15	Not reported	Not reported	Not reported	Healthy	35	Not reported	Not reported	Not reported	20 active CD<br>15 remission CD
37	Mortensen	2017	PLoS one	Denmark	Case control	Y	N	Rome III	22	20	34.4 (16-75)	Mean	Not reported	24	3	41.3 (19-60)	Mean	Healthy	132	45CD<br>31 UC	35.8 (15-76) CD<br>37.3 (21-70)	Mean	72 CD<br>60 UC
38	Thorsvik	2017	Journal of Gastroenterology and Hepatology	Norway	Case control	Y	N	Rome III	21	17	42 (22-95)	Median	Not reported	23	16	40 (22-71)	Median	17 Healthy<br>6 outpatient	94	22 UC<br>18 CD<br>12 IEC	43 (19-76) UC<br>37 (17-69) CD<br>41 (22-95) IEC	Median	43 UC<br>30 CD<br>21 Infectious enterocolitis
39	Aksoy	2018	San Paulo Medical Journal	Turkey	Case control	Y	N	Rome III	28	18	48.2±11.7	Mean	Not reported	15	7	41.4±12.6	Mean	Healthy	39	15	46.1±12.6	Mean	UC
40	Du	2018	Digestive Diseases and Sciences	China	Case control	Y	N	Rome III	112	59	45.6±12.7	Mean	112 IBS-D	46	24	43.1±11.4	Mean	Healthy	0	0	0	0	0

41	Guo	2018	International Journal of Clinical and Experimental Medicine	China	Case control	Y	N	Rome III	68	20	45.4±4.80	Mean	68 IBS-C	68	32	41.24±2.81	Mean	Healthy	0	0	0	0	0
42	Liu	2018	World Journal of Gastroenterology	China	Case control	Y	N	Rome III	42	15	29.4 (22-40)	Mean	42 IBS-D	20	8	28.9 (20-38)	Mean	Healthy Asymptomatic	0	0	0	0	0
43	Russo	2018	Disease Markers	Italy	Case control	Y	N	Rome III	34	30	41.2±2.1	Mean	34 IBS-D	17	12	39.5±2.9	Mean	Healthy	0	0	0	0	0
44	Sahin-Erylmaz	2018	Turkish Journal of Gastroenterology	Turkey	Case control	Y	N	Rome III	60	25 IBS-C 10 IBS-D	42.47±10.40 IBS-C 39.87±11.05 IBS-D	Mean	30 IBS-C 30 IBS-D	30	17	48.60±14.79	Mean	Asymptomatic	0	0	0	0	0
45	Weaver	2018	Nursing Research	USA	Case control	Y	N	Rome	5	5	29.4±9.3	Mean	5 IBS-C	5	5	31.8±4.9	Mean	Healthy	0	0	0	0	0
46	Hayatbakhsh	2019	Reports of Biochemistry and Molecular Biology	Iran	Case control	Y	N	Rome III	96	43	31.7±11.54 males 65.6±11.32 females	Mean	32 IBS-D 33 IBS-C 31 IBS-M	44	20	31.8±10.95 males 32.16±12.82 females	Mean	Healthy	0	0	0	0	0
47	Morales	2019	Gastroenterology	Not reported	Not reported	Y	N	Not reported	84	Not reported	Not reported	NA	84 IBS-D	0	0	0	0	0	31	Not reported	Not reported	NA	22 UC 9 CD
48	Singh	2019	United European Gastroenterology Journal	USA	Case control	Y	N	Rome III	100	12 IBS-D 48 IBS-C	32.1±12 IBS-D 31.6±10.5 IBS-C	Mean	50 IBS-C 50 IBS-D	42	33	35.2±10.5	Mean	Healthy	53	35	41.2±16.6	Mean	Coeliac disease
49	Tian	2019	Journal of Gastroenterology and Hepatology	China	Case control	Y	Y	Rome IV	21	8	31.64±8.85	Mean	21 IBS-D	14	6	27±4.38	Mean	Healthy	0	0	0	0	0
50	Zhang	2019	World Journal of Gastroenterology	China	Case control	N	Y	Rome IV	30	8	30.0 (28.0, 41.3)	Median (IQR)	30 IBS-D	15	4	28.0 (25.0, 37.0)	Median (IQR)	Healthy	0	0	0	0	0
51	Zhu	2019	Chinese Medical Journal	China	Case control	Y	N	Rome III	120	Not reported	Not reported	NA	120 IBS-D	144	Not reported	Not reported	NA	Healthy	0	0	0	NA	0
52	Aktas	2020	Family Medicine and Primary Care Review	Turkey	Retrospective review	Y	N	Rome IV	87	60	40 (20)	Median	Not reported	61	35	38 (13)	Median	Healthy (outpatient checkups?)	0	0	0	NA	0

53	Barbaro	2020	Gut	Italy	Case control	Y	N	Rome III	59	31	43.5±18.2	Mean	59 IBS-D	25	16	33.0±7.5	Mean	Healthy Asymptomatic outpatient	101	71 NCGS 12 CD	39.0±12.6 NCGS 37.5±14.3	Mean	86 NCGS 15 CD
54	Chira	2020	Bosnian Journal of Basic Medical Sciences	Romania	Case control	Y	N	Rome III	40	29	55.36±14.80	Mean	20 IBS-C 14 IBS-D 6 IBS-M	33	17	57.93±11.49	Mean	Healthy	33	14	49.78±16.65	Mean	Colorectal cancer
55	Eltayeb	2020	Parkistan Journal of Biological Sciences	Sudan	Cross sectional	Y	N	Rome III	40	24	Not reported	NA	18 IBS-D 8 IBS-C 14 IBS-M	40	19	Not reported	NA	Healthy	0	0	0	NA	0
56	Gur	2020	Nobel Medicus	Turkey	Cross sectional	N	Y	Rome III	121	59	39.5±13.8	Mean	Not reported	0	0	0	NA	0	272	88 UC 45 CD	43.1±13.9 UC 38.1±13.3 CD	Mean	182 UC 90 CD
57	Lee	2020	Neurogastroenterology and Motility	Korea	Case control	N	Y	Rome IV	29	16	38.9±9.7	Mean	29 IBS-D	22	10	37.4±10.1	Mean	Healthy	0	0	0	NA	0
58	Xu	2020	Frontiers in Cellular and Infection Microbiology	China	Case control	Y	Y	Rome III	22	10	40.7±3.1	Mean	22 IBS-D	15	10	44.8±2.9	Mean	Healthy	15	9	42.3±3.0	Mean	Depression
59	Zhang	2020	Neurogastroenterology and Motility	China	Case control	Y	N	Rome IV	177	123	39.12±15.23	Mean	77 IBS-D 27 IBS-C 11 IBS-M 30 IBS-U 32 PI-IBS	174	124	40.56±14.28	Mean	Healthy	0	0	0	NA	0
60	Bilooka	2021	Journal of Medicine and Life	Ukraine	Case control	Y	N	Not reported	37	Not specified	38.56±2.59 IBS-D 35.56±2.27 IBS-C	Mean	18 IBS-D 19 IBS-C	21	Not specified	32.81±1.36	Mean	Healthy	10	Not specified	32.00±2.19	Mean	Obesity
61	Borekci	2021	Turkish Journal of Biochemistry	Turkey	Case control	Y	N	Rome IV	75	32 IBS-C 9 IBS-D 12 IBS-M	43.14±14.18 IBS-C 39.85±10.22 IBS-D 46.69±12.93 IBS-M	Mean	49 IBS-C 13 IBS-D 13 IBS-M	79	45	40.84±10.81	Mean	Healthy	0	0	0	NA	0
62	de-Faria	2021	Scandinavian Journal of Gastroenterology	Sweden	Case control	Y	N	Rome III	78	69	30 (18-56)	Median	18 IBS-D 17 IBS-C 43 IBS-M	40	36	32 (20-55)	Median	Healthy	20	12	56 (32-76)	Median	Coeliac disease

63	Gacesa	2021	Gut Microbes	Netherlands	Case control	N	Y	Rome III	169	118	44 (29, 58)	Median (IQR)	30 IBS-C<br>61 IBS-D<br>69 IBS-M	1044	553	46 (35, 56)	Median (IQR)	Healthy	447	264	44 (33, 56)	Median (IQR)	250 CD<br>168 UC<br>29 IBD unknown
64	James	2021	Metabolites	New Zealand	Case control	N	Y	Rome IV	105	23 IBS-C<br>40 IBS-D<br>24 IBS-M	53.5 IBS-C<br>52.IBS-D<br>50.5 IBS-M	Mean	24 IBS-C<br>52 IBS-D<br>29 IBS-M	97	52	54.4	Mean	Asymptomatic outpatients	0	0	0	NA	0
65	Makhlouf	2021	Egyptian Journal of Hospital Medicine	Egypt	Case control	Y	Y	Not reported	10	Not reported	Not reported	Not reported	Not reported	10	Not reported	Not reported	Not reported	Healthy	30	Not reported	Not reported	Not reported	Numbers not specified
66	McEvoy	2021	United European Gastroenterology Journal	Australia	Case control (nested in cohort study)	Y	N	Rome III	156	96	64.5±6.5	Mean	55 IBS-D<br>24 IBS-C<br>65 IBS-M<br>12 IBS-U	332	166	65.2±7.1	Mean	Population	0	0	0	NA	0
67	Mokhtar	2021	Acta Gastro-Enterological Belgica	Malaysia	Case control	Y	N	Rome III	77	23	27 (20-49)	Median (IQR)	77 IBS-C	88	50	28 (20-50)	Median (IQR)	Healthy	0	0	0	NA	0
68	Norlin	2021	Brain, Behaviour and Immunity	Sweden	Case control	Y	N	Rome III	88	75	30.0 (11.0)	Median (IQR)	25 IBS-D<br>16 IBS-C<br>47 IBS-M	47	40	31.0 (19.0)	Median (IQR)	Healthy	0	0	0	NA	0
69	Barandouzi	2022	Scientific Reports	USA	Case control	Y	N	Rome IV	40	26	21.67±2.22	Mean	Not reported	20	10	20.10±1.41	Mean	Healthy	0	0	0	NA	0
70	Casado-Bedmar	2022	Journal of Leukocyte Biology	Sweden	Case control	N	Y	Rome III	37	37	27 (21-47)	Median (range)	21 IBS-M<br>8 IBS-D<br>8 IBS-C	20	Not reported	Not reported	NA	Healthy	0	0	0	NA	0
71	de Graaf	2022	Nutrients	Netherlands	Case control	N	Y	Rome III	261	195	43.3±17.0	Mean	56 IBS-C<br>93 IBS-D<br>103 IBS-M<br>9 IBS-U	195	123	44.4±18.9	Mean	Healthy	238	126	45.7±14.8	Mean	156 CD<br>82 UC
72	Edwinson	2022	Nature Microbiology	USA	Case control	N	Y	Rome III	52	Not reported	Not reported	Not reported	52 PI-IBS	30	Not reported	Not reported	Not reported	Healthy	0	0	0	NA	0

73	Guven	2022	Turkish Journal of Gastroenterology	Turkey	Case control	Y	N	Rome IV	107	67	46.6±15.2	Mean	107 IBS-C	107	58	45.3±13.3	Mean	Asymptomatic outpatients	0	0	0	NA	0
74	Han	2022	ISME Journal	China	Case control	Y	Y	Rome IV	264	149	43.99±11.23	Mean	24 IBS-C<br>215 IBS-D<br>19 IBS-M<br>7 IBS-U	66	48	40.32±11.95	Mean	Asymptomatic outpatients	0	0	0	NA	0
75	Ismail	2022	Annals of Parasitology	Iraq	Case control	Y	N	Not reported	250	Not reported	Not reported	Not reported	150 Blastocystis +ve<br>100 Blastocystis -ve	100	Not reported	Not reported	Not reported	78 Healthy<br>22 Blastocystis +ve	0	0	0	NA	0
76	Liu	2022	Chinese Medical Journal	China	Case control	Y	N	Rome III	127	35	32.61±9.68	Mean	127 IBS-D<br>51 lactulose, hydrogen, and methane breath test +ve<br>76 lactulose, hydrogen, and methane breath test -ve	49	16	32.28±9.68	Mean	BT -ve, Healthy	0	0	0	NA	0
77	Monem	2022	Journal of Medicine and Life	Egypt	Case control	Y	N	Rome IV	32	18	43.53±13.38	Mean	16 IBS-C<br>16 IBS-D	16	5	47±11.61	Mean	Healthy	0	0	0	NA	0
78	Mujagic	2022	Gut Microbes	Netherlands	Case control	Y	Y	Rome III	181	126	44.7±16.9	Mean	64 IBS-D<br>34 IBS-C<br>73 IBS-M<br>10 IBS-U	133	78	45.8±18.9	Mean	Healthy	0	0	0	NA	0
79	Paljetak	2022	Gut Microbes	Croatia	Case control	Y	Y	Rome III	26	15	31 (19-56)	Median (range)	Not reported	12	6	35 (24-56)	Median (range)	Healthy	23	7 CD<br>6 UC	46 (21-72) CD<br>31 (18-54) UC	Median (range)	10 CD<br>13 UC
80	Rezazadegan	2022	Scientific Reports	Iran	Case control	Y	N	Rome IV	61	38	37.01±8.46	Mean	61 IBS-D	61	38	37.32±9.02	Mean	Healthy	0	0	0	NA	0

81	Rezazadegan	2022	Digestive Diseases and Sciences	Iran	Case control	Y	N	Rome IV	61	38	37.01±8.46	Mean	61 IBS-D	61	38	37.32±9.02	Mean	Healthy	0	0	0	NA	0
82	Rydell	2022	Journal of Clinical Medicine	Sweden	Case control	Y	Y	Rome IV	100	71	36 (18-57)	Medan (range)	Not reported	0	0	0	NA	0	97	26 UC<br>20 CD	38 (18-79) UC<br>39 (20-78) CD	Median (range)	59 UC<br>38 CD
83	Wang	2022	Digestive Diseases and Sciences	Netherlands	Longitudinal cohort	N	Y	Rome IV	91	72	41.7 ± 14.4	Mean	26 IBS-C<br>32 IBS-D<br>19 IBS-M<br>14 IBS-U	30	26	39.4 ± 16.9	Mean	Healthy	0	0	0	NA	0
84	James	2023	Metabolites	New Zealand	Case control	Y	N	Rome IV	60	19 IBS-C<br>32 IBS-D	Not reported	Not reported	20 IBS-C<br>40 IBS-D	72	37	Not reported	Not reported	Asymptomatic outpatients	0	0	0	NA	0
85	Thomas-Dupont	2023	Journal of Clinical Gastroenterology	Mexico	Case control	Y	N	Rome III	400	335	44.7±2	Mean	124 IBS-C<br>220 IBS-M<br>56 IBS-D	400	335	*44.5±2*	Mean	Asymptomatic outpatients	0	0	0	NA	0
86	Yao	2023	Digestive Diseases and Sciences	China	Case control	Y	N	Rome IV	120	71	41.672±11.740	Mean	120 IBS-D	63	38	36.921±11.83	Mean	Healthy	0	0	0	NA	0
87	Ohman	2011	European Journal of Gastroenterology and Hepatology	Sweden	Case control	Y	N	Rome II	74	52	34±16	Mean	26 IBS-D<br>11 IBS-C<br>37 IBS-M	30	20	39±10	Mean	Healthy	0	0	0	NA	0
88	Bednarska	2017	Gastroenterology	Sweden	Case control	Y	N	Rome III	37	37	32.2 (19-55)	Mean (range)	21 IBS-M<br>8 IBS-C<br>8 IBS-D	20	20	29.9 (20-48)	Mean (range)	Healthy	0	0	0	NA	0
89	Gao	2013	Experimental and Therapuetic Medicine	China	Case control	Y	N	Rome III	28	16	39 (17-61) normal psych<br>37.5 (17-58) anxiety/dep	Mean (range)	28 IBS-D	15	9	38.5 (20-57)	Mean (range)	Polyps and hemorrhoids	0	0	0	NA	0
90	Hanevik	2022	Journal of Gastroenterology and Hepatology	Norway	Cohort	Y	N	Rome II	31	24	41.3±11.3	Mean	14 IBS-D	41	25	40.4±12.4	Mean	Healthy	22	18	42.4±9.5	Mean	CFS

91	Chojnacki	2022	Polski merkuriusz lekarski : organ Polskiego Towarzystwa Lekarskiego	Poland	Case control	Y	Y	Rome IV	50	32	69.6±6.3	Mean	50 IBS-U	50	17	68.6±7.4	Mean	Healthy	0	0	0	NA	0
92	Acharya	2023	Euroasian Journal of Hepatogastroenterology	India	Case control	Y	Y	Rome III	25	8	34.92±8.87	Mean	25 IBS-D	0	0	0	NA	0	50	18	38.46±12.12	Mean	50 UC
93	Darkoh	2014	PLoS one	USA	Case control	Y	Y	Rome II	60	13 PI-IBS<br>36 non-PI IBS	50 (28-66)	Mean (range)	16 PI-IBS<br>44 non-PI IBS	40	23	53 (25-86)	Mean (range)	0	0	0	NA	0	0
94	Dinan	2006	Gastroenterology	Ireland	Case control	Y	N	Rome II	76	50	34.6±13.1	Mean	30 IBS-M<br>36 IBS-D<br>10 IBS-C	75	50	30.2±13.5	Mean	0	0	0	0	NA	0
95	Kindt	2009	Neurogastroenterology and Motility	Belgium	Case control	Y	N	Rome II	30	24	39.0 (30.0-45.0)	Range	Not reported	32	25	30.5 (24.5-44.5)	Range	Healthy	0	0	0	NA	0
96	Lembo	2009	Alimentary Pharmacology and Therapeutics	USA	Case control	Y	N	Rome II	876	718	38 (18-35 years)<br>29 (36-50 years)<br>29 (>50 years)	Distribution	297 IBS-C<br>272 IBS-D<br>289 IBS-M<br>18 IBS-U	235	136	45 (18-35 years)<br>35 (36-50 years)<br>20 (>50 years)	Distribution	Healthy	455	Not reported for iBD/CD	Not reported for iBD/CD	Not reported for iBD/CD	398 IBD<br>57 Coeliac
97	Liebregts	2007	Gastroenterology	Australia	Case control	Y	N	Rome II	55	33	39.5	Median	18 IBS-M<br>17 IBS-C<br>20 IBS-D	36	23	37.5	Median	0	0	0	0	NA	0
98	Pimentel	2015	PLoS one	USA	Case control	Y	N	Rome III	2375	1606	44.4±12.2	Mean	2375 IBS-D	43	29	36.0±9.9	Mean	Healthy	263	79 IBD<br>92 Coeliac	40.9±11.7 IBD<br>41.6±12.3 coeliac	Mean	73 CD<br>69 UC<br>121 Coeliac
99	Rana	2012	Tropical Gastroenterology	India	Case control	Y	N	Rome II	63	26	42.6±19.5	Mean	63 IBS-D	62	30	43.5±18.7	Mean	Healthy	0	0	0	NA	0
100	Sundin	2018	Scientific Reports	Sweden	Case control	N	Y	Rome III	88	54	35 (28-44)	Median (IQR)	27 IBS-D<br>31 IBS-C<br>30 IBS-U	33	21	27 (25-35)	Median (IQR)	Healthy	0	0	0	NA	0
101	Semnani	2009	Journal of Clinical Gastroenterology	Iran	Case control (nested in cohort study)	Y	N	Rome II	80	46	31.83± 10.89	Mean	80 IBS-M	80	49	32.38 ± 13.55	Mean	Healthy	0	0	0	NA	0

102	Seyedmirzaee	2016	Clinics and Research in Hepatology and Gastroenterology	Iran	Case control	Y	N	Rome III	74	46	35.52±11.72	Mean	34 IBS-D<br>29 IBS-C<br>11 IBS-M	75	48	37.37±12.5	Mean	Healthy	0	0	0	NA	0
103	Chojnacki	2024	International Journal of Molecular Sciences	Poland	Case control	Y	Y	Rome IV	36	29	44.7±11.3	Mean	36 IBS-M	36	28	45.4±9.4	Mean	Healthy	0	0	0	NA	0
104	Huong	2024	Medical Archives	Vietnam	Case control	Y	Y	Rome IV	215	112	48.7±13.2	Mean	215 IBS-D	31	16	46.1±11.9	Mean	Healthy	89	54	44.2±14.5	Mean	71 UC<br>18 CD
105	Rezaaie	2017	Digestive Diseases and Sciences	USA	Case control	Y	N	Rome III	2430	1606 IBS-D<br>16 IBS-M<br>24 IBS-C	44.4±12.2 IBS-D<br>40.1±13.7 IBS-M<br>40.5±14.0 IBS-C	Mean	2375 IBS-D<br>25 IBS-M<br>30 IBS-C	43	29	36.0±9.9	Mean	Healthy	0	0	0	NA	0
106	Sarhan	2023	Egyptian Journal of Internal Medicine	Egypt	Case control	Y	Y	Rome IV	291	206	35.5±8	Mean	42 IBS-C<br>49 IBS-M<br>137 IBS-U<br>63 IBS-D	20	Not reported	Not reported	Not reported	Not reported	51	35	39±8.7	Mean	51 UC
107	Talley	2020	Gut	Australia	Cohort	Y	N	Rome III	86	Not reported	Not reported	NA	18 IBS-D clinic<br>14 IBS-D population	182	Not reported	Not reported	NA	Healthy	26	Not reported	Not reported	NA	5 coeliac<br>6 IBD<br>5 GORD
108	Teige	2024	Neurogastroenterology and Motility	Norway	Case control	N	Y	Rome IV	60	46	38.0±11.4	Mean	21 IBS-D<br>31 IBS-M<br>8 IBS-C	42	27	35.62±12.3	Mean	Healthy	0	0	0	NA	0
109	Ekoff	2024	Clinical and Translational Gastroenterology	Sweden	Cross sectional	Y	Y	Rome IV	100	71	36 (29.3-52.5)	Median (IQR)	Not reported	0	0	0	NA	0	100	26 UC<br>20 CD	38 (27.0-54.0) UC<br>38.5 (28.8-64.0) CD	Median (IQR)	59 UC<br>38 CD<br>1 microscopic colitis<br>2 unclear IBD
110	Schmulson	2016	Revista de Gastroenterologia de Mexico	Mexico	Cross sectional	Y	N	Rome III	20	13	34 (16-65) IBS-D<br>43 (26-56) IBS-D overlap<br>45 (27-75) IBS-M	Median (range)	17 IBS-D<br>3 IBS-M<br>7 PI-IBS<br>12 non-PI IBS	0	0	0	NA	0	0	0	0	NA	0

111	Talley	2019	Clinical and Translational Gastroenterology	Australia	Cohort	Y	N	Rome III	319	Not reported		NA	97 IBS-D clinic<br>60 IBS-C clinic	246	Not reported	Not reported	NA	Healthy	129	Not reported	Not reported	NA	7 coeliac disease<br>41 IBD<br>74 other
112	Ahmed	2024	Journal of Clinical and Diagnostic Research	India	Cross sectional	N	Y	Rome IV	90	Not reported	42.2	mean	65 IBS-D<br>22 PI-IBS<br>15 IBS-C<br>8 IBS-M<br>2 IBS-U	0	0	0	NA	0	40	Not reported	Not reported	NA	Not reported
113	Huang	2024	Journal of Inflammation Research	China	Case control	Y	N	Rome IV	86	53	39.9±10.6	Mean	86 IBS-D	106	64	38.5±10.2	Mean	Healthy	0	0	0	NA	0
114	Ramio-Pujol	2020	GastroHep	Spain	Case control	N	Y	Rome IV	52	37	48.2±13.6	Mean	35 IBS-D<br>5 IBS-M<br>3 IBS-C	61	39	44.8±14.0	Mean	Healthy	52	13 CD<br>14 UC	51.0±17.7 CD<br>53.9±15.4 UC	Mean	25 CD<br>27 UC
115	Gargari	2023	Gut Microbes	Italy	Case control	N	Y	Rome IV	240	148	36±12	Mean	136 IBS-D<br>104 IBS-M	100	54	39±13	Mean	Healthy	0	0	0	NA	0
116	Al-Khafaji	2025	Cellular and Molecular Biology	Iraq	Case control	Y	N	Rome IV	50	50	20-65	Range	Not reported	25	25	20-65	Range	Healthy	0	0	0	NA	0
117	Al-Shammari	2025	Journal of Applied Hematology	Iraq	Case control	Y	N	Rome IV	50	27	33.18±7.45	Mean	Not reported	50	25	35.3±6.52	Mean	Healthy	0	0	0	NA	0
118	Barros	2024	BMC Gastroenterology	Brazil	Case control	Y	Y	Rome IV	45	35	40±14.5	Mean	45 IBS-D	46	33	56.6±11.6	Mean	Healthy	44	20	39.7±14.1	Mean	24 UC<br>20 CD
119	Kumar	2024	Indian Journal of Gastroenterology	India	Case control	N	Y	Rome III	56	14	37.79±13.59	Mean	56 IBS-D	0	0	0	NA	0	57	15	38.42±13.9	Mean	32 CD<br>24 UC<br>1 indeterminate
120	Mohamed	2025	Egyptian Journal of Medical Microbiology	Egypt	Case control	Y	Y	Rome IV	55	32	37.55±7.51	Mean	27 IBS-D<br>21 IBS-C<br>7 IBS-M	26	13	36.62±5.70	Mean	Healthy	0	0	0	NA	0
121	Roth	2024	BMC Gastroenterology	Sweden	Case control	Y	N	Rome IV	260	212	44 (33.25-56)	Mean (range)	90 IBS-M<br>46 IBS-C<br>70 IBS-D<br>10 IBS-U<br>41 FBD	50	37	37.85 (30.18-45.48)	Mean (range)	Healthy	0	0	0	NA	0

122	Tuncel	2025	PeerJ	Turkey	Case control	Y	N	Rome IV	57	Unclear as reported	50.41±15.88 IBS-D 54.61±13.89 IBS-C	Mean	28 IBS-C 29 IBS-D	28	Unclear as reported	58.00±13.89	Mean	Healthy	0	0	0
123	Venge	2025	Neurogastroenterology and Motility	Sweden	Case control	N	Y	Rome III	165	114	46 (20-73)	Median (range)	31 IBS-C 58 IBS-D 76 non C/D	44	22	44 (18-73)	Median (range)	Healthy	0	0	0
124	Matijasic	2025	Journal of Molecular Medicine	Croatia	Case control	Y	Y	Rome III	23	13	32 (19-56)	Median	Not reported	12	6	35 (24-56)	Median (range)	Healthy	26	9 CD 7 UC	45 (21-72) CD 32 (18-54) UC

Row		
122	NA	0
123	NA	0
124	Median (range)	13 CD 13 UC

Supplementary Methods: Code used in the metafor package in rstudio for meta-analysis (1)

```
#Data table must look like Mean1, Mean2, SD1, SD2, N1, N2 e.g.

#                          N1     N2     Mean1         Mean2        SD1
        SD2
#Author1, Year1            14     10     1.786  6.5     0.28         1.118
#Author2, Year2     76     115     3.03   6.23         1.75         3.5

#Use metafor package for meta-analysis
install.packages("metafor")
library(metafor)
library(tidyverse)

##################################################################
#Load excel file for meta analysis (must be a csv file) #

Factor <- read.table(file="file_name.csv", header=TRUE, sep = ",")

####### calculate effect size from mean and SD########
Factor <- escalc(measure="SMD", m1i=Mean1, m2i=Mean2, sd1i=SD1, sd2i=SD2, n1i=N1,
n2i=N2, data= Factor)

##metafor will add in effect size (yi) and variance (vi)
View(Factor)

####### meta-analysis using a random-effects model #######
res_ Factor <- rma(yi, vi, data= Factor method="REML")
res_ Factor

#######Forest plot ########
# X-axis limits
min_val <- min(res_Factor $yi - 1.96 * sqrt(res_Factor $vi))
max_val <- max(res_Factor $yi + 1.96 * sqrt(res_Factor $vi))
padding <- 3
xlim <- c(min_val - padding, max_val + padding)

# Study labels
study_labels <- paste(Factor$Author..year, sep = ", ")
total_IBS <- sum(Factor$N1)
total_control group <- sum(Factor$N2)

# Auto-scale plot dimensions
n_studies <- length(res_Factor$yi)
max_label_length <- max(nchar(study_labels))
plot_height <- max(6, n_studies * 0.8)
plot_width  <- max(17, 6 + max_label_length * 0.2)
```

```
# Auto-title filename from plot title
plot_title <- "FactorName"
safe_title <- gsub("[^a-zA-Z0-9]", "_", plot_title)
filename <- paste0(safe_title, ".svg")

# Open SVG device
svg(filename, width = plot_width, height = plot_height)

# Set margins BEFORE plotting
par(mar = c(5, 4, 4, 2) + 0.1)

# Create forest plot
forest(res_Factor,
       xlab = "Standardized Mean Difference",
       annotate = TRUE,
       header = "Study",
       showweights = TRUE,
       main = plot_title,
       xlim = xlim,
       digits = c(2, 1),
       slab = study_labels,
       cex = 2.0,
       cex.main = 2.0,
       addfit = TRUE,
       boxsize = 0.08)

# Close device
dev.off()

##Explaining heterogeneity with meta regression ##
##moderator analyses ##
# Add additional data for moderators

total_participants <- Factor$N1 + Factor$N2

Factormoderator <- c('')
Factor$moderator <- Factormoderator


# Meta-regression
res.Factorn <- rma(yi, vi, mods = ~ total_participants, data=Factor)
res.Factorn

res.Factormoderator <- rma(yi, vi, mods = ~ Factormoderator, data=Factor)
res.Factormoderator


## Testing for Publication Bias ###
#Funnel plot
funnel (res_Factor)
```

```
#Egger's test of funnel plot asymmetry
regtest(res_Factor)

#The trim and fill method (imputes "missing" studies to create a more symmetrical funnel plot)
res.tf <- trimfill(res_Factor)
res.tf

#fail-safe N
fsn(yi,vi,data=Factor, type="Rosenthal")

## To test for influential studies##
##baujat plot to identify influential studies
baujat (res_Factor)

##tests for influential studies
inf <- influence(res_Factor)
inf

#### Generates report of all results including figures ######
reporter(res_Factor)

##############################################################################################
```

1. Viechtbauer W. Conducting Meta-Analyses in R with the metafor Package. Journal of Statistical Software. 2010;36(3):1 - 48.

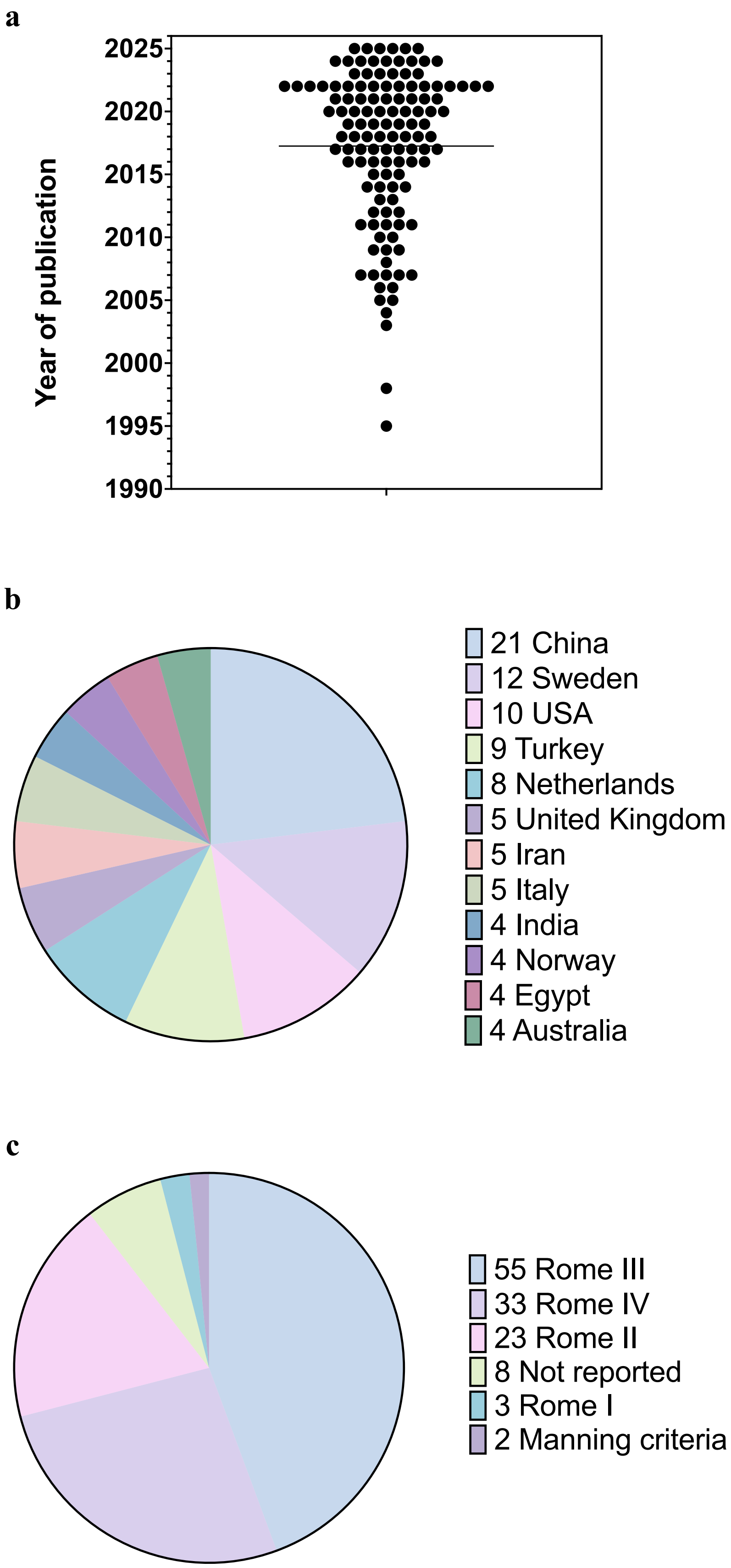

Supplementary Figure 1: Characteristics of studies included in this systematic review.
(A) Publication year for all 124 included studies. Line denotes median. (B) Number of studies included by country (list of top 10 out of n=33 countries in total, n=4 countries tied for 10th posiiton). (C) Pie chart of the diagnostic criteria used by all included studies to define the IBS cohort

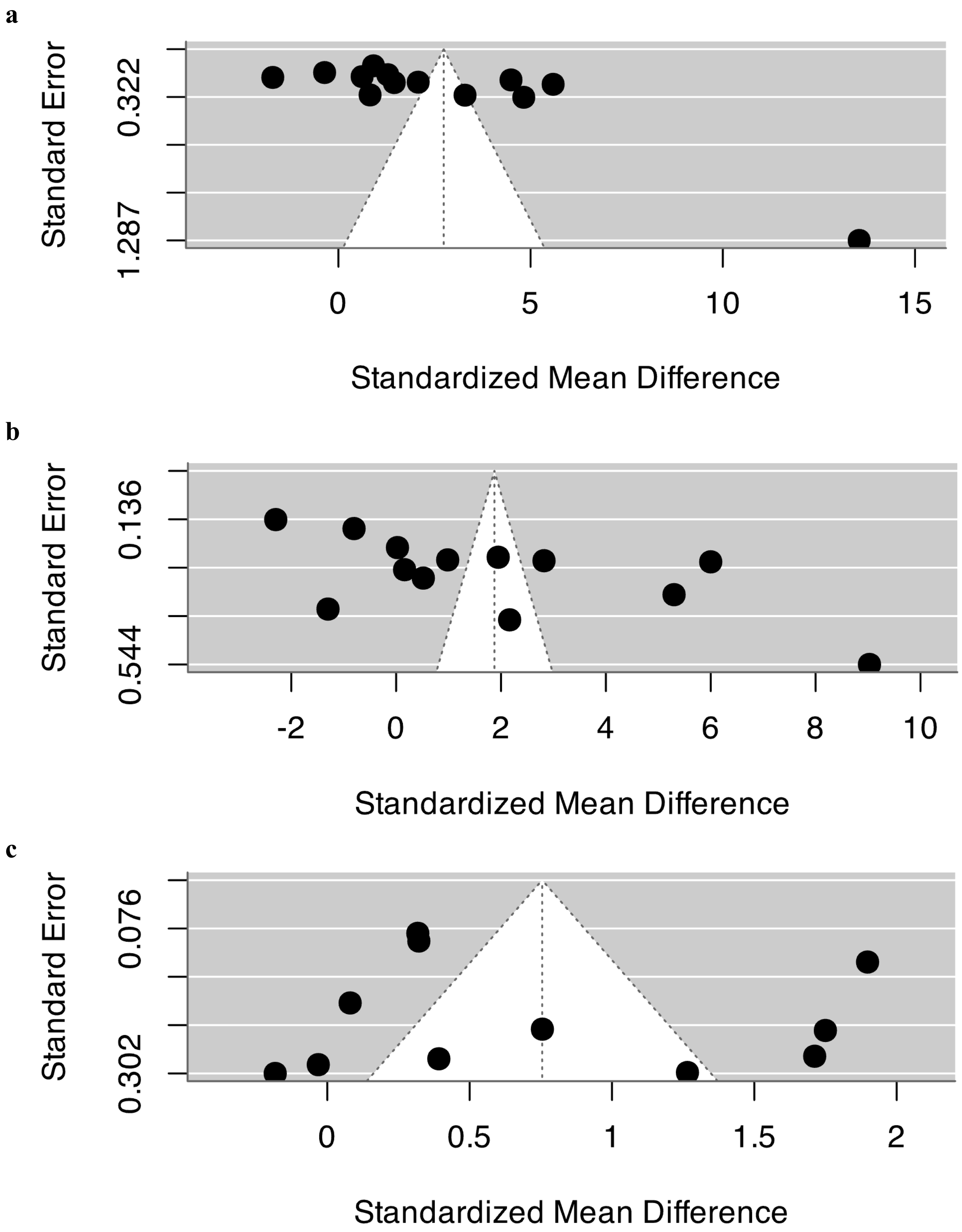

Supplementary Figure 2: Funnel plots for studies with 10 or more studies assessing IBS compared to healthy or asymptomatic control subjects.

Funnel plots and Egger's regression test were used to assess for possible publication bias in meta-analysed factors with greater than n=10 included studies where the SMD was significantly different between IBS and controls for peripheral factors including (A) TNF-α (n=13 studies), (B) IL-6 (n=13 studies) and (C) faecal calprotectin (n=11 studies)

a

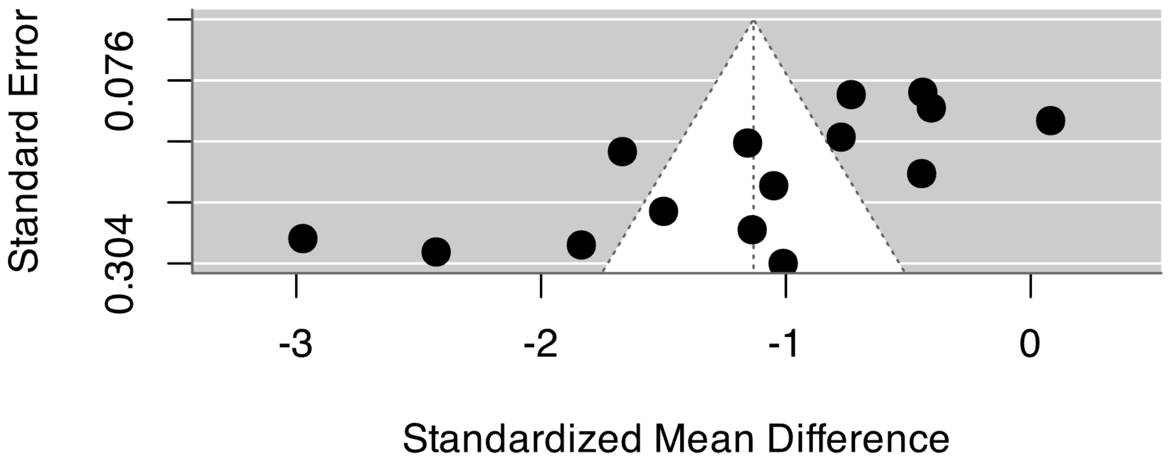

Supplementary Figure 3: Funnel plot for studies assessing faecal calprotectin in subjects with IBS compared to those with organic gastrointestinal diseases.
Funnel plots and Egger's regression test were used to assess for possible publication bias in studies examining faecal calprotectin in IBS compared to organic disease subjects (n=16 studies total).
